# PS-Net: human perception-guided segmentation network for EM cell membrane

**DOI:** 10.1093/bioinformatics/btad464

**Published:** 2023-07-28

**Authors:** Ruohua Shi, Keyan Bi, Kai Du, Lei Ma, Fang Fang, Lingyu Duan, Tingting Jiang, Tiejun Huang

**Affiliations:** Advanced Institute of Information Technology, Peking University, Hangzhou, Zhejiang 310000, China; National Engineering Research Center of Visual Technology, National Key Laboratory for Multimedia Information Processing, School of Computer Science, Peking University, Beijing 100871, China; Beijing Academy of Artificial Intelligence, Beijing 100084, China; School of Psychological and Cognitive Sciences and Beijing Key Laboratory of Behavior and Mental Health, Peking University, Beijing 100871, China; IDG/McGovern Institute for Brain Research, School of Psychological and Cognitive Sciences, Peking University, Beijing 100871, China; Key Laboratory of Machine Perception (Ministry of Education), Peking University, Beijing 100871, China; Peking-Tsinghua Center for Life Sciences, Peking University, Beijing 100084, China; Institute for Artificial Intelligence, Peking University, Beijing 100871, China; National Engineering Research Center of Visual Technology, National Key Laboratory for Multimedia Information Processing, School of Computer Science, Peking University, Beijing 100871, China; Beijing Academy of Artificial Intelligence, Beijing 100084, China; National Biomedical Imaging Center, College of Future Technology, Peking University, Beijing 100871, China; School of Psychological and Cognitive Sciences and Beijing Key Laboratory of Behavior and Mental Health, Peking University, Beijing 100871, China; IDG/McGovern Institute for Brain Research, School of Psychological and Cognitive Sciences, Peking University, Beijing 100871, China; Key Laboratory of Machine Perception (Ministry of Education), Peking University, Beijing 100871, China; Peking-Tsinghua Center for Life Sciences, Peking University, Beijing 100084, China; National Engineering Research Center of Visual Technology, National Key Laboratory for Multimedia Information Processing, School of Computer Science, Peking University, Beijing 100871, China; Peng Cheng Laboratory, Shenzhen 518066, China; Advanced Institute of Information Technology, Peking University, Hangzhou, Zhejiang 310000, China; National Engineering Research Center of Visual Technology, National Key Laboratory for Multimedia Information Processing, School of Computer Science, Peking University, Beijing 100871, China; National Engineering Research Center of Visual Technology, National Key Laboratory for Multimedia Information Processing, School of Computer Science, Peking University, Beijing 100871, China; Beijing Academy of Artificial Intelligence, Beijing 100084, China

## Abstract

**Motivation:**

Cell membrane segmentation in electron microscopy (EM) images is a crucial step in EM image processing. However, while popular approaches have achieved performance comparable to that of humans on low-resolution EM datasets, they have shown limited success when applied to high-resolution EM datasets. The human visual system, on the other hand, displays consistently excellent performance on both low and high resolutions. To better understand this limitation, we conducted eye movement and perceptual consistency experiments. Our data showed that human observers are more sensitive to the structure of the membrane while tolerating misalignment, contrary to commonly used evaluation criteria. Additionally, our results indicated that the human visual system processes images in both global–local and coarse-to-fine manners.

**Results:**

Based on these observations, we propose a computational framework for membrane segmentation that incorporates these characteristics of human perception. This framework includes a novel evaluation metric, the perceptual Hausdorff distance (PHD), and an end-to-end network called the PHD-guided segmentation network (PS-Net) that is trained using adaptively tuned PHD loss functions and a multiscale architecture. Our subjective experiments showed that the PHD metric is more consistent with human perception than other criteria, and our proposed PS-Net outperformed state-of-the-art methods on both low- and high-resolution EM image datasets as well as other natural image datasets.

**Availability and implementation:**

The code and dataset can be found at https://github.com/EmmaSRH/PS-Net.

## 1 Introduction

**Figure 1.**
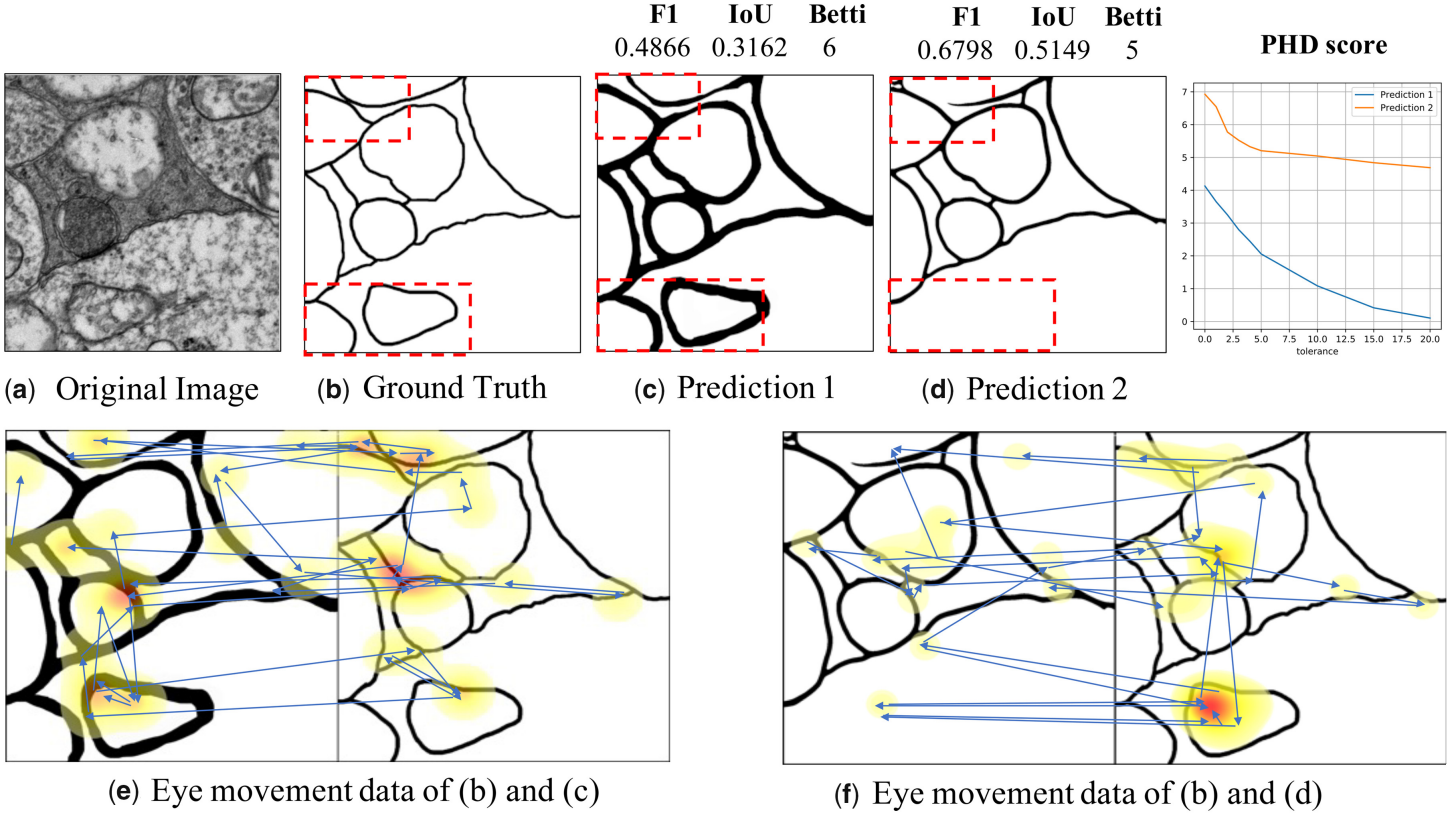
Illustrations of subjective experiments and eye movement experiments. (a and b) Original EM image with its ground truth of cell membranes. (c and d) Cell membrane segmentation predictions of (a). The boxes indicate the errors of (d). (e) Eye movement data of subjects when comparing (b) and (c). (f) Eye movement data of subjects when comparing (b) and (d). The heatmaps show the accumulated time of fixations, and the arrows show the directions of the saccades.

Electron microscopy (EM) techniques are widely used to study the ultrafine structures of biological tissues at the nanometer scale (Curry *et al.* 2006, Harris *et al.* 2006, Erlandson 2009). One important task in EM image analysis is the segmentation of cell membranes, which has numerous applications including reconstructing neural connections (Fakhry *et al.* 2016) and visualizing cell morphology (Pelling *et al.* 2005, Pallotto *et al.* 2015). Compared to the semantic segmentation task of natural images, membrane segmentation of EM images is more challenging because of its higher resolution, more complex structure, and more details (Fig. 1a).

Despite the significant progress made by deep-learning (DL) methods (Ronneberger *et al.* 2015, Paszke *et al.* 2016, Chaurasia and Culurciello 2017, Shen *et al.* 2017, Yu *et al.* 2017, Hu *et al.* 2018, Khadangi *et al.* 2021) in the segmentation of EM cell membrane segmentation [ISBI 2012 (Arganda-Carreras *et al.* 2015)] approaching or even surpassing human performance, their performance has been observed to deteriorate on high-resolution datasets (both in terms of the absolute number of pixels and the size of each pixel). Specifically, DL methods have achieved ∼98% accuracy (V-Rand) on the ISBI 2012 dataset, but only about 60% on the high-resolution U-RISC dataset (Shi *et al.* 2022) with 10×10k pixels. In contrast, human performance on both datasets has been found to be consistently high (close to 99%). This led us to question why human vision is more robust than DL when dealing with images with complex textures and contours. In this study, we aim to investigate the mechanisms of the human visual system for the EM image segmentation task and identify the underlying causes for these differences in performance.

During our investigation into the differences in performance between humans and DL methods on cell membrane segmentation, we noticed that there is a discrepancy between human perception and commonly used evaluation criteria, such as the *F*1 score (Sasaki *et al.* 2007), IoU (Kosub 2019), and Betti number error (Betti) (Hu *et al.* 2021). For example, in Fig. 1, (b) is the ground truth of the cell membranes in (a), while (c) and (d) are two predictions by different algorithms. According to the *F*1, IoU, and Betti scores, prediction (d) is better than prediction (c). However, from a human perspective, the opposite is true because (d) lacks some important structures. To better understand this discrepancy, we conducted a subjective experiment in which subjects were shown three images: the ground truth and two different predictions. They were asked to indicate which prediction was more similar to the ground truth. We evaluated the consistency between the preferences of these criteria and humans. Surprisingly, results showed that these evaluation criteria are only 30%–40% consistent with human perception.

To better understand the mechanisms of the human visual system when comparing two images of cell membranes, we conducted an eye movement experiment to record subjects’ saccades and fixations. In the experiment, subjects were shown two images of cell membranes side by side, such as the ground truth (b) and prediction (c) in Fig. 1e. Heatmaps and arrows were used to indicate fixations and saccades, respectively. Based on the data collected from eye movements, we found that humans focus primarily on the structure of membranes while using quick glances to compare other regions. For example, the red regions of the heatmaps correspond to junctions of cell membranes in (e) and missing edges in (f). This suggests that humans pay more attention to the skeleton of the cell membrane and missing edges, while ignoring thickness and misalignment errors. Additionally, we observed that humans use a global-local strategy and a coarse-to-fine approach to find differences. Specifically, according to the saccades, we found that humans first roughly scan the images to locate areas with different structures and then repeatedly compare these areas carefully. These observations are consistent with the concept of spatial-frequency (multiscale) analysis in neuroscience (Spillmann 1999, Beaucousin *et al.* 2013, Nayar *et al.* 2015), which suggests that the biological human visual system employs a global–local strategy for processing images. Other studies (Hegdé 2008, Flevaris *et al.* 2014) have also found that visual processing follows a coarse-to-fine progression, which helps to quickly process high-resolution images.

Based on the insights gained from our investigations, we present a cell membrane segmentation system that is designed to conform to human visual perception. Specifically, we propose a new evaluation metric called the Perceptual Hausdorff Distance (PHD), which measures the dissimilarity of cell membranes by extracting their skeletons as point-sets and calculating the distance with a flexible tolerance distance that simulates human tolerance for minor errors. Our experiments showed that the PHD metric is more consistent with human perception than other evaluation criteria. Additionally, we design an end-to-end trainable network called the PHD-guided segmentation network (PS-Net) for the segmentation of high-resolution EM images, which takes into account both local and global features using a structure extraction module. During training, we use a newly proposed PHD loss with an adaptive weight, which is based on the PHD metric, to simulate the coarse-to-fine processing characteristic of the human visual system.

To evaluate the effectiveness of our new system, we compare it with state-of-the-art methods on the EM image datasets ISBI 2012 and U-RISC. The results show that our proposed PS-Net outperforms existing methods on all evaluation criteria. We also demonstrate the versatility of our method by applying it to natural image segmentation datasets, where it also demonstrates state-of-the-art performance.

## 2 Related works

**EM cell membrane segmentation**, which can also be viewed as cell boundary detection, is a critical step in EM image analysis for neuron reconstruction. This task is more challenging than similar tasks on natural images, such as “delineation detection,” due to the higher resolution, more complex structures, and more detailed information present in EM images. Since the release of the first annotated EM image dataset in the ISBI 2012 challenge (Arganda-Carreras *et al.* 2015), several extraordinary DL methods have been developed for this task. U-Net (Ronneberger *et al.* 2015) is a popular and successful DL model for biomedical image segmentation. Subsequent research efforts (Paszke *et al.* 2016, Chaurasia and Culurciello 2017, Shen *et al.* 2017, Yu *et al.* 2017, Hu *et al.* 2018, Khadangi *et al.* 2021) have sought to further improve EM segmentation performance using a U-shaped encoder–decoder architecture and effective feature extraction techniques, such as dual-channel blocks (Lou *et al.* 2021) and skip connections (Chaurasia and Culurciello 2017). These methods have achieved near-human performance on the ISBI 2012 dataset. However, as EM imaging techniques have advanced, the demand for the segmentation of ultra-high-resolution images has increased. For instance, the recently proposed U-RISC dataset (Shi *et al.* 2022) has a resolution of 120×9958×9959. When applied to this dataset, the performance of these methods significantly decreased (from 98% on ISBI 2012 to 60% on U-RISC). This suggests that algorithms should not only focus on effectively extracting features from limited labeled images, but should also incorporate human-based strategies.

**Evaluation for cell membrane segmentation.** In the cell membrane segmentation task, both pixel accuracy and topographic accuracy are important. There are three main categories of evaluation criteria (Yeghiazaryan and Voiculescu 2018) that have been proposed for image segmentation: “pixel-wise” criteria, “topology-wise” criteria, and “point-wise” criteria. “Pixel-wise” criteria, such as the *F*1 and IoU, treat segmentation as a pixel-wise binary classification task and use statistics to evaluate the performance of models. These criteria are often used as optimization objectives, with popular loss functions including the cross-entropy loss and its variations (Chen *et al.* 2019, Khadangi *et al.* 2021), as well as the Dice loss (Dice 1945). “Topology-wise” criteria, like V-Rand and V-Info (Arganda-Carreras *et al.* 2015) consider both merge and split errors of membranes in their evaluation. Betti (Hu *et al.* 2021) is another topology-wise criterion that compares the topology (number of handles) of the predicted and ground truth boundaries. The recently proposed clDice (Shit *et al.* 2021) modifies the Dice by skeletonization to gain the topology sensitivity. However, these criteria can be complex and may be affected by small split errors. “Point-wise” criteria, such as Hausdorff distance and its variations, measure the difference between the predicted and ground truth boundaries using distance metrics. However, these criteria may not be adaptive to different scales and human tolerance for small misalignment. Our eye movement experiments suggest that humans are not only concerned with pixel accuracy, but also tolerant of small errors. Therefore, in this work, we aim to design a criterion with high consistency with human perception and develop an end-to-end network for high-resolution EM image segmentation.

## 3 Perceptual consistency experiment and eye movement experiment

To verify whether existing evaluation criteria for cell membrane segmentation are consistent with human perception, we conducted a perceptual consistency experiment and eye movement experiments. The results of these experiments were used to explore the attentional mechanisms of the human visual system when observing EM segmentation results. An example in the Fig. 1 illustrates the inconsistency between human perception and three popular criteria, including *F*1, IoU, and Betti, leading us to question the suitability of these criteria for evaluating the cell membrane segmentation task.

### 3.1 Perceptual consistency experiment

We selected six state-of-the-art segmentation methods and applied them to the U-RISC dataset, generating 200 groups of segmentation predictions. Each group contained three images: the ground truth image in the center and two segmentation predictions for the same input cell image on either side (the interface is shown in Supplementary Section S1). Twenty subjects participated in the experiment and were asked to identify which of the two segmentation results was closest to the truth. Our results showed that pixel-wise criteria, such as the *F*1 score and IoU had a consistency of only 34.51% and 35.40%, respectively, while the topology-wise criterion, the Betti, had a consistency of 47.78%. These findings suggest that these criteria do not align well with human subjective perception. Further details on the experimental setup can be found in Supplementary Section S1.

### 3.2 Eye movement experiment

We randomly selected 50 groups of images from a dataset used in a previous experiment on human subjective perception. Each group contained two images: a prediction and the ground truth of the same input cell image (as shown in Fig. 1e). The eye movement data were collected from 20 subjects using the EyeLink 1000 Plus with a high-speed camera at a 2000 Hz sampling rate. Subjects were asked to identify the differences between the two images. During the experiment, the images were presented in a random order and the saccades and fixation maps were recorded. We found that subjects focused more on the structures of the membranes and were tolerant of small misalignments. Additionally, the saccades of subjects indicated that they used a global–local strategy and coarse-to-fine approach, first scanning the image roughly to locate areas with different structures and then carefully comparing those regions repeatedly. These findings suggest that for cell membrane segmentation tasks, humans would use a global–local strategy and coarse-to-fine manner. More fixation maps and saccades are shown in Supplementary Section S2.

## 4 Perceptual Hausdorff distance

The results of subjective experiments indicated that the commonly used evaluation criteria for natural image segmentation were not in alignment with human perception of cell membrane segmentation. Meanwhile, the eye movement experiment provided insight into how humans visually compare images of cell membranes, leading to the development of a new evaluation criterion based on human perception, known as PHD.

In the design of the PHD, we consider the structural information of cell membranes and the human tolerance for slight misalignment. On the one hand, to capture the structural information, we represent membranes as point-sets and use the modified Hausdorff distance (Huttenlocher *et al.* 1993). As the results of eye movement experiments show, humans are more sensitive to changes in structure than to changes in thickness of membranes. Therefore, to alleviate the influence of the thickness change, the PHD extracts structural information (skeleton) from the segmentation results and represents it as a point-set. Then, it calculates the distance between two point-sets using an modified version of the Hausdorff distance (Huttenlocher *et al.* 1993), which averages the shortest distance between the two point-sets rather than taking the maximum value to better reflect global information. This allows PHD to be more robust to outliers and better aligned and pay more attention to the global information.

**Figure 2.**
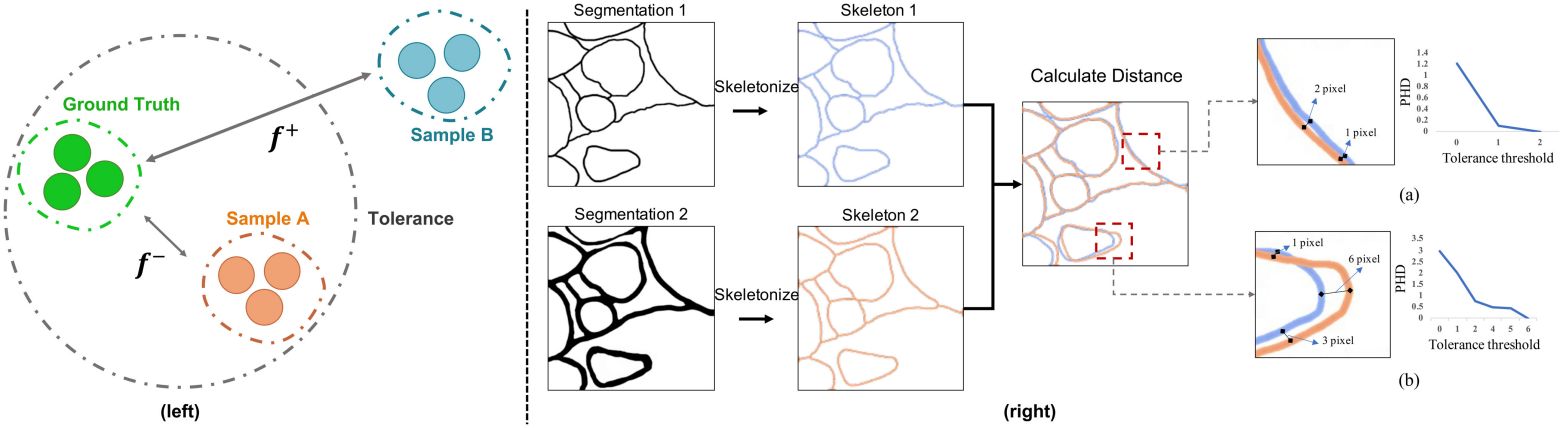
Overview of PHD. (Left) Illustration of the tolerance of human vision. (Right) Evaluation with PHD. PHD takes two segmentation results as input. Then, the two inputs are skeletonized. Finally, a PHD distance can be calculated between two skeletons with different tolerance thresholds. (a) and (b) are two cases for intuitively understanding the influence of tolerance distance in PHD.

On the other hand, to account for human tolerance, we define the concept called “Tolerance Distance” Ψ(x,y) [Equation (1)], between two points as a function of the Euclidean distance d(x,y) between them and a threshold value τ representing the tolerance for small misalignment errors. We use the rectified linear unit (ReLU) to adjust the distance depending on whether it exceeds the threshold. As Fig. 2 (left) shows, if d(x,y)>τ, it is magnified by $f^{+}$, otherwise, it is narrowed by $f^{-}$. Considering the calculation cost and subjective consistency, we use $f^{+}(d)=d$ and $f^{-}(d)=0$ in the following experiments, which is similar to the ReLU. Ablation studies for $f^{+}$ and $f^{-}$ are shown in Supplementary Section S4.5.


(1) $$\Psi (x,y)=\{\begin{array}{cc} f^{+}(d(x,y)), & d(x,y)>\tau \\ f^{-}(d(x,y)), & d(x,y)\leq \tau \end{array}.$$

To summarize, given unordered non-empty point-sets *X*, *Y*, and the tolerance distance Ψ(x,y), the PHD is defined as Equation (2).


(2) $$d_{\text{PHD}}(X,Y)=\frac{1}{\left|X\right|}\sum_{x\in X}\underset{y\in Y}{\min}\Psi (x,y)+\frac{1}{\left|Y\right|}\sum_{y\in Y}\underset{x\in X}{\min}\Psi (x,y).$$

The tolerance distance, represented by the parameter τ, determines the level of tolerance for small misalignment errors in the PHD metric. This is demonstrated in Fig. 2, which shows two examples of the influence of the tolerance distance on the PHD value. In the first example (a), the two skeletons (shown in blue and orange) are close in Euclidean space but not coincident. When τ=0, indicating no tolerance for errors, the PHD value is high. As τ increases, the PHD value decreases. In the second example (b), there is a large offset between the two skeletons. When τ is set to values within the range [2, 4], the decline in the PHD value is slow. It only drops to 0 when τ=6, which is the maximum distance between the two skeletons.

### 4.1 Consistency between PHD and human perception

**Figure 3.**
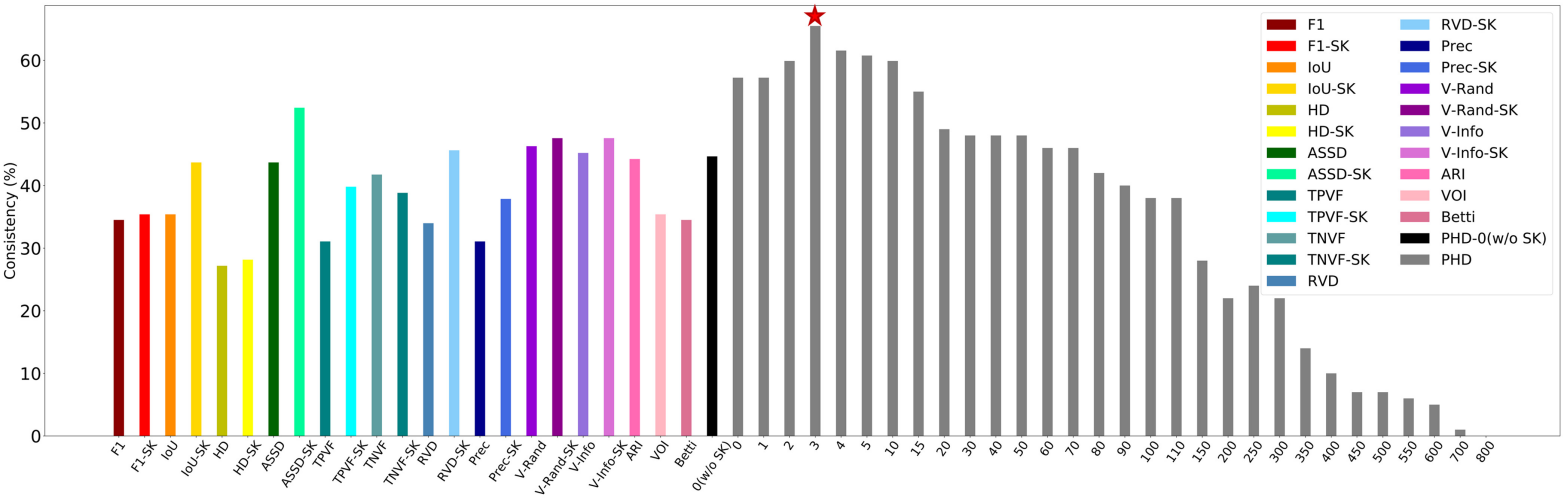
Consistency with human perception. The left color bars show the consistency results of varies of criteria. −SK represents the metrics with skeletonization. The gray bars show the results of PHD with different threshold τ (the numbers on *X*-axis). And the black bar shows the PHD without skeletonization (w/o SK) with tolerance distance equal to 0.

Our experiments have shown that PHD is consistent with human perception and can effectively evaluate the performance of cell membrane segmentation algorithms. As shown in Fig. 3, in comparison to 14 popular metrics (color bars) including *F*1 (Sasaki *et al.* 2007), clDice (Shit *et al.* 2022), IoU (Kosub 2019), Hasudorff (Huttenlocher *et al.* 1993), ASSD (Yeghiazaryan and Voiculescu 2018), TPVF (Yeghiazaryan and Voiculescu 2018), TNVF (Yeghiazaryan and Voiculescu 2018), RVD (Yeghiazaryan and Voiculescu 2018), Precision (Yeghiazaryan and Voiculescu 2018), V-Rand (Arganda-Carreras *et al.* 2015), V-Info (Arganda-Carreras *et al.* 2015), ARI (Weng *et al.* 2021), VOI (Weng *et al.* 2021), and Betti (Hu *et al.* 2021), PHD (gray bars) showed higher consistency with human perception. The results of different tolerance thresholds in PHD and the use of skeletonization (−SK) for other metrics were also compared. Our findings indicate that PHD is a useful tool for evaluating cell membrane segmentation algorithms. The formulas are shown in Supplementary Section S3.

Based on the results of our experiments, it was found that the criterion of PHD demonstrated the highest level of consistency with the human perception among the popular evaluation metrics tested. In particular, the best consistency between PHD and human perception was 65.48% when the tolerance threshold was set to 3. This is nearly double the consistency scores obtained by the *F*1 score or IoU, and significantly higher than the scores for V-Rand and V-Info. Additionally, as the tolerance threshold for PHD increased from 0 to 800, the consistency with human perception initially increased before slowly decreasing to 0, indicating that humans do have tolerance for a certain level of offset. These results suggest that humans tend to tolerate small perturbations in cell membrane segmentation.

It is worth investigating whether using skeletonization can improve the performance of other evaluation metrics. The results in Fig. 3 show that using skeletonization can help some metrics, such as F1, IoU, and ASSD, to a certain extent. However, the consistency of *F*1−SK is only 34.51%, and the consistency of IoU−SK is 44.25%. These values are still far from the performance of PHD. This suggests that simply extracting the membrane skeleton is not sufficient to address the limitations of existing metrics.

## 5 PHD-guided segmentation network

Inspired by the PHD criterion and the global–local strategy with a coarse-to-fine approach observed in the eye movement experiment, we propose the PS-Net. This network includes a multiscale architecture with loss functions specifically designed to guide the segmentation process using PHD.

### 5.1 Overview of architecture

**Figure 4.**
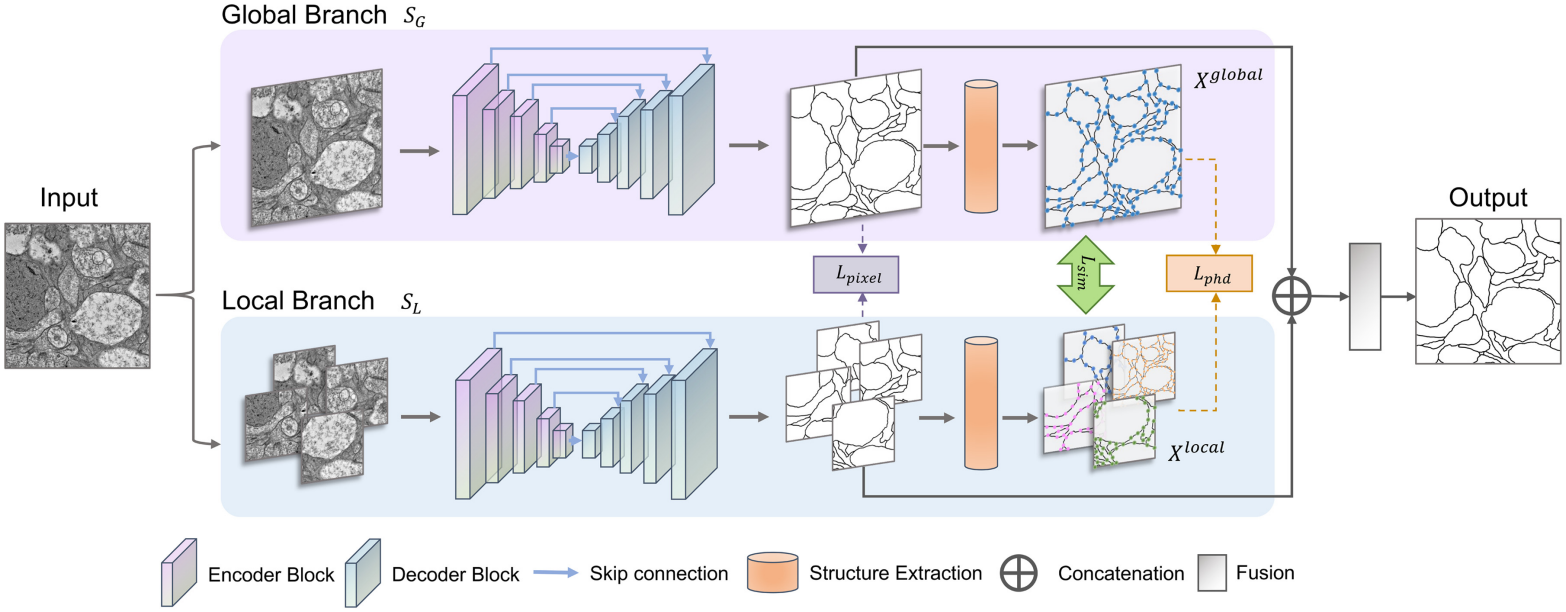
An overview of PS-Net. PS-Net has two branches to segment multiple scales of the input image. In the global branch, the u-shape segmentation module uses the original image as input and outputs its membrane probability map. In the local branch, the original image is cropped into *N* patches with the same size. Then, the patches are put into the segmentation module with *N* prediction maps as outputs. The two branches share weights during the training process. The structure extraction module is designed to compute the skeletons of the all the *N* predictions. Three loss functions: pixel-wise loss, PHD loss, and similarity loss are calculated during the training. PS-Net outputs the prediction from the results of two branches.

An overview of the network is depicted in Fig. 4. PS-Net consists of two branches for multiscale image segmentation: the “global branch” $S_{G}$, which uses the full image as input, and the local branch $S_{L}$, which uses *N* patches of the cropped original image with the same size as input. Both branches use the same u-shaped encoder–decoder architecture to make probability predictions, as well as a module for skeleton extraction. The global and local predictions are then combined to produce the final segmentation result.

#### 5.1.1 Backbone

The U-Net (Ronneberger *et al.* 2015) is a convolutional neural network with a contracting path that captures contextual information and an expansive path that enables precise localization. It is often used as an encoder–decoder module in image segmentation tasks. In this work, the U-Net is utilized in the global and local branches of the PS-Net for probability prediction.

#### 5.1.2 Skeleton extraction module

The structure extraction module of PS-Net uses the modified differentiable Zhang–Suen thinning algorithm (Zhang and Suen 1984) to extract the skeleton of the membrane from the binary output of the soft-max layer (details in Supplementary Section S7). This algorithm is fast and reliable for media-axis extraction, and has shown to be stable in obtaining the skeleton of images, even when the number of pixels occupied by the membrane is small. Other thinning methods, such as distance transform (Felzenszwalb and Huttenlocher 2012), were also considered but were found to be less stable in these cases.

#### 5.1.3 Fusion module

This model generates the point-sets of the membranes ($X^{\mathrm{global}}$ of global branch and $X^{\mathrm{local}}$ of local branch), which are used to assist the pixel-wise segmentation during the training process. The final prediction is obtained by combining the results of two branches and employing an average pooling layer with the probability maps as inputs.

### 5.2 Loss functions

As for loss function design, different from previous methods (Chen *et al.* 2019, Yan *et al.* 2021), which focus on pixel-wise accuracy, PS-Net considers both pixel-wise and topology-wise accuracy. Specifically, three loss functions are utilized during the training process: pixel-wise loss $L_{\mathrm{pixel}}$, PHD loss $L_{\mathrm{phd}}$, and similarity loss $L_{\mathrm{sim}}$. $L_{\mathrm{pixel}}$ [Equation (3)] is the combination of the WCE loss function (Ronneberger *et al.* 2015) and Dice loss (Drozdzal *et al.* 2016), which measures the local similarity of prediction and ground truth in both global and local branches. where *M* is the number of pixels of the image, *C* is the number of classes, which is two in this task. $g_{i}^{c}$ is a binary indicator if class label *c* is the correct classification for pixel *i*, and $s_{i}^{c}$ is the corresponding predicted probability. $w_{c}$ is the reciprocal of the class frequency in the training set.


(3) $$L_{\mathrm{pixel}}=1-\frac{2\sum_{i=1}^{M}\sum_{j=1}^{C}g_{i}^{c}s_{j}^{c}}{\sum_{i=1}^{M}g_{i}^{c}+\sum_{i=1}^{M}s_{i}^{c}}-\frac{1}{M}\sum_{i=1}^{M}\sum_{j=1}^{C}\omega_{c}g_{i}^{c}\;\text{log}\;s_{j}^{c},$$

The second loss function is the PHD loss $L_{\mathrm{phd}}$, which is used to penalize the difference in membrane structures between the ground truth and predictions in both the global and local branches. As shown in Equation (4), the function compares the skeleton point-sets of the predictions, represented by $X^{\mathrm{global}}$ and $X^{\mathrm{local}}$, with their respective ground truth, represented by $Y^{\mathrm{global}}$ and $Y^{\mathrm{local}}$. In order to compute the loss for backpropagation, the soft-max function is applied to the likelihood map for binarization and the derivative of the binary image is shown in Supplementary Section S7.


(4) $$L_{\mathrm{phd}}=d_{\mathrm{PHD}}(X^{\mathrm{global}},Y^{\mathrm{global}})+d_{\mathrm{PHD}}(X^{\mathrm{local}},Y^{\mathrm{local}}).$$

In addition, the similarity loss $L_{\mathrm{sim}}$ is used to measure the similarity between the global and local scales by calculating the PHD distance between the skeleton point-sets of the global prediction $(X^{\mathrm{global}}$ and the stitched local predictions ${\hat{X}}^{\mathrm{local}})$. It is designed as $L_{\mathrm{sim}}=d_{\mathrm{PHD}}(X^{\mathrm{global}},{\hat{X}}^{\mathrm{local}})$. The stitched local predictions ${\hat{X}}^{\mathrm{local}}$ are obtained by stitching the skeleton point-sets $X^{\mathrm{local}}$ from the local branch, and have the same size as the global skeleton point-sets $X^{\mathrm{global}}$. This loss helps to ensure that the prediction from the global branch and the stitched prediction from the local branch are consistent in terms of structure.

### 5.3 Coarse-to-fine training

During the training process, three loss functions are optimized with a coarse-to-fine strategy, which aims to assist the network focusing more on generating a coarse segmentation result, and then subsequently shifting to detailed information. Correspondingly, in our method, $L_{\mathrm{pixel}}$ measures the accuracy of each pixel in the image, which is the low-level (local) feature, while $L_{\mathrm{phd}}$ and $L_{\mathrm{sim}}$ measure the structural difference of membranes, which is the high-level (global) feature. In contrast to the two-stage refinement approach utilized by Chen *et al.* (2019), PS-Net employs pixel-wise loss for the first several epochs as to generate a coarse segmentation result. And then, to get a finer cell membrane structure, the weights of PHD loss and similarity loss are adaptively raised with the number of training epochs. Let $\lambda_{1}$ and $\lambda_{2}$ be the adaptive weights of $L_{\mathrm{phd}}$ and $L_{\mathrm{sim}}$. The final loss function of PS-Net *L* is shown in Equation (5). The details of the parameter settings are shown in Supplementary Section S4.2.


(5) $$L=L_{\mathrm{pixel}}+\lambda_{1}L_{\mathrm{phd}}+\lambda_{2}L_{\mathrm{sim}}.$$

## 6 Segmentation experiments

The performance of PS-Net was evaluated on two EM image datasets. Results show that PS-Net outperforms existing methods. Then, ablation studies were performed to isolate the individual contributions of the main components and parameters of our approach. Furthermore, PS-Net was extended to two natural image segmentation datasets with SOTA performance.

### 6.1 Experiments on EM image datasets

We evaluated our method on two EM datasets: ISBI 2012 and U-RISC. We used a 3-fold cross-validation to tune hyperparameters for both our proposed method and eight baseline methods. The evaluation metrics included *F*1 score, IoU, V-Rand, V-Info, TPVF, TNVF, Hausdorff distance, and our proposed PHD-τ, where τ is the tolerance threshold. The baseline methods included U-Net (Ronneberger *et al.* 2015), CASENet (Yu *et al.* 2017), LinkNet (Chaurasia and Culurciello 2017), GLNet (Chen *et al.* 2019), SENet (Hu *et al.* 2018), U-Net++ (Zhou *et al.* 2018), Mosin. (Mosinska *et al.* 2018), and DMT (Hu *et al.* 2021). We report the mean and SD performance over the test set for all the methods. More details about the datasets, baseline models, and evaluation metrics are provided in Supplementary Section S4. * represents that the predicted results for evaluation are re-implemented by the official code.

**Figure 5.**
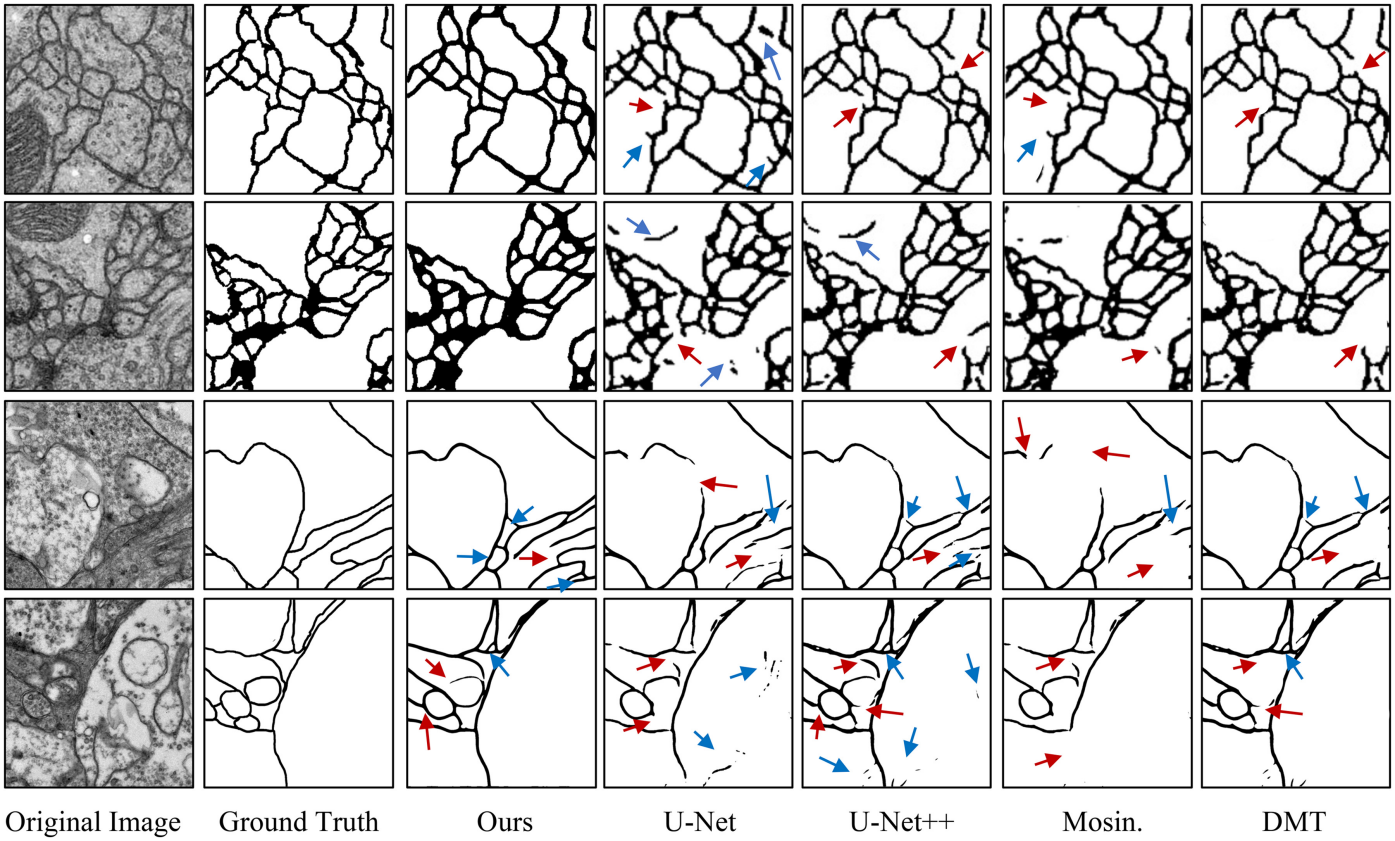
Segmentation results of ISBI 2012 (first two rows) and U-RISC (last two rows) datasets. Red arrow: false negative error. Blue arrow: false positive error.

For the ISBI 2012 dataset, our method achieves SOTA performance as reported in Table 1. We also summarized some leading quantitative results reported in original papers in Supplementary Sections S4.3 and S4.4. The results show that PS-Net obtained the best scores on all of these metrics (as shown in bold font). More visualizations of segmentation results are depicted in Fig. 5. Our method has fewer mistakes. More visualization results are shown in Supplementary Section S4.7.

**Table 1.** Quantitative results of the methods on ISBI 2012 dataset.

Metrics	U-Net*	CASENet*	LinkNet*	GLNet*	SENet*	U-Net++*	Mosin.*	DMT*	PS-Net
*F*1 (%)	92.01±0.02	87.99±0.05	89.40±0.04	90.41±0.02	91.35±0.02	93.01±0.02	82.30±0.03	92.93±0.02	**93.98** ± **0.02**
IoU (%)	92.31 ±0.01	89.61±0.01	91.02±0.01	81.89±0.02	84.24±0.01	89.56±0.02	90.88±0.01	92.19±0.01	**93.99** ± **0.01**
V-Rand (%)	96.33±0.02	96.53±0.32	96.99±0.04	95.69±0.07	94.54±0.04	95.81±0.05	95.99±0.04	96.74±0.06	**98.37** ± **0.02**
V-Info (%)	96.01±0.02	96.27±0.03	95.01±0.04	96.56±0.02	96.42±0.01	97.07±0.05	95.81±0.05	97.82±0.01	**98.75** ± **0.02**
TNVF (%)	94.61±0.01	93.12±0.02	93.08±0.03	93.52±0.02	92.04±0.03	94.25±0.03	94.66±0.01	94.67±0.02	**94.68** ± **0.01**
TPVF (%)	91.96±0.04	91.77±0.05	89.94±0.07	91.80±0.04	90.49±0.03	91.45±0.02	92.04±0.03	92.75±0.02	**93.00** ± **0.04**
ASSD ↓	2.689±1.92	3.157±1.13	3.921±2.15	3.036±2.01	3.002±1.19	2.994±2.05	3.015±1.87	3.845±1.86	**2.041** ± **1.98**
HD ↓	55.94±10.4	59.87±17.0	63.12±28.1	83.12±17.0	72.46±19.4	60.35±10.5	93.03±19.2	84.94±13.6	**54.62** ± **13.8**
PHD-0 ↓	5.950±2.06	6.013±1.05	5.814±4.52	6.989±3.57	5.362±2.50	4.205±3.95	4.833±2.97	4.374±3.19	**3.954** ± **1.04**
PHD-3 ↓	5.650±2.07	5.990±1.02	5.627±4.66	6.884±3.10	5.028±2.18	4.002±3.65	4.629±2.54	4.081±3.02	**3.661** ± **1.25**
PHD-5 ↓	3.663±1.91	4.280±3.82	3.631±0.82	5.299±3.84	3.716±2.11	3.769±3.02	3.894±1.18	3.351±2.44	**3.042** ± **1.53**
PHD-10 ↓	2.414±1.08	2.997±2.05	2.146±1.07	3.017±2.58	2.631±1.98	2.877±1.94	2.510±1.01	1.993±2.31	**1.045** ± **0.99**
PHD-50 ↓	0.280±0.01	0.241±0.03	0.351±0.02	0.26±0.017	0.291±0.02	0.238±0.03	0.274±0.03	0.286±0.04	**0.244** ± **0.01**

The boldface values indicate the best performance.

For the U-RISC dataset, we first summarize the top four results reported in the leaderboard of the challenge (Supplementary Section S4.4). Our method has reached the best performance (promote approximately **11.5%** more than the winning team in the challenge). Similar to the experiments of ISBI 2012, to compare more results of other metrics, we train and test the six competitive methods, using the data division the same as the challenge. The scores and SD of eight evaluation metrics are reported on the testing images. The results in Table 2 show that PS-Net outperformed the other methods. In particular, it not only greatly improves the *F*1 score, but also performs well in other metrics. In addition, we observed an apparent decline of the PHD-τ scores at τ=10 and τ=50 for ISBI 2012 and U-RISC, respectively, which showed that the U-RISC was a more challenging dataset to gain a fine segmentation. Compared with other methods, our method is able to alleviate the missing structures and redundant predictions (as shown in Supplementary Section S4.8).

**Table 2.** Quantitative results of the methods on U-RISC dataset.

Metrics	U-Net*	CASENet*	LinkNet*	GLNet*	SENet*	U-Net++*	Mosin.*	DMT*	PS-Net
*F*1 (%)	48.83±0.02	60.07±0.05	60.70±0.04	58.10±0.04	52.12±0.05	60.30±0.05	47.56±0.09	39.68±0.05	**67.69** ± **0.02**
IoU (%)	32.33±0.02	43.07±0.05	**43.69** ± **0.05**	41.05±0.04	35.41±0.05	43.29±0.04	40.29±0.08	37.98±0.06	43.63±0.03
V-Rand (%)	49.38±0.03	59.21±0.05	63.10±0.04	53.41±0.04	52.88±0.05	62.11±0.04	49.75±0.05	50.37±0.04	**68.93** ± **0.02**
V-Info (%)	51.20±0.04	60.13±0.04	62.39±0.03	54.33±0.04	51.78±0.06	62.34±0.04	58.64±0.03	59.27±0.05	**65.32** ± **0.03**
TNVF (%)	88.62±0.02	96.22±0.05	96.02±0.03	95.72±0.04	97.68±0.05	95.92±0.03	94.25±0.05	96.31±0.04	**97.82** ± **0.02**
TPVF (%)	35.24±0.03	56.04±0.04	55.62±0.04	53.39±0.04	52.91 ±0.04	54.93±0.04	54.99±0.03	53.77±0.05	**56.17** ± **0.03**
ASSD ↓	10.16±8.14	9.314±3.51	9.201±4.43	11.96±9.45	12.11±6.34	9.106±4.52	19.67±10.3	13.04±8.45	**7.808** ± **4.15**
HD ↓	271.5±31.1	566.1±32.2	352.9±29.9	399.3±39.1	547.3±38.0	414.0±31.9	484.6±51.5	683.9±82.4	**252.8** ± **30.2**
PHD-0 ↓	18.65±9.72	19.25±9.33	22.72±6.93	23.30±6.46	20.42±5.22	17.25±7.33	24.54±8.98	29.56±9.57	**15.29** ± **5.79**
PHD-3 ↓	17.93±8.52	19.01±10.2	22.70±6.92	23.15±6.05	19.86±6.13	16.99±8.21	24.26±7.38	29.56±8.48	**15.01** ± **6.29**
PHD-5 ↓	17.37±6.25	16.72±9.15	20.41±7.81	21.25±5.74	17.47±10.0	16.55±7.01	22.85±8.62	28.78±10.3	**13.52** ± **5.03**
PHD-10 ↓	8.512±5.10	10.38±6.99	11.90±8.66	11.53±6.03	9.93±7.23	8.99±6.72	19.48±7.29	18.67±6.27	**6.979** ± **6.67**
PHD-50 ↓	6.501±1.17	10.25±5.64	5.436±2.82	5.170±4.62	3.201±2.53	4.312±2.97	15.47±6.13	14.52±6.29	**1.594** ± **2.06**

The boldface values indicate the best performance.

### 6.2 Ablation study on U-RISC

To evaluate the effectiveness of the proposed two strategies and three loss functions, we conducted several ablation experiments on the U-RISC dataset.

#### 6.2.1 PHD-based loss functions

To evaluate the effectiveness of PHD-based loss functions, we trained the model using only the pixel-wise loss $L_{\mathrm{pixel}}$, and then added the PHD loss $L_{\mathrm{phd}}$ and similarity loss $L_{\mathrm{sim}}$. The results are presented in Table 3, where $L_{1}$, $L_{2}$, and $L_{3}$ represent $L_{\mathrm{pixel}}$, $L_{\mathrm{phd}}$, and $L_{\mathrm{sim}}$, respectively. The results show that $L_{\mathrm{phd}}$ improved the performance of the three architectures. In particular, the *F*1 score increased by ∼6.63% and the PHD-0 score decreased by ∼1.83 when using $L_{\mathrm{phd}}$. Additionally, the combination of $L_{\mathrm{sim}}$ with $L_{\mathrm{phd}}$ resulted in an ∼1.49% increase in the *F*1 score and a decrease of ∼0.78–1.176 in the PHD score. This indicates that the structure of the cell membrane plays an important role in its segmentation. Furthermore, for the selection of the tolerance, we conducted the ablation experiments summarized in Supplementary Table S5. The results show that PS-Net achieves the best performance when τ=2. Further, we also compared the PHD loss with another topology loss, clDice loss (Shit *et al.* 2022), in Supplementary Table S6, and the results verify the superiority of PHD loss.

**Table 3.** Ablation study for the architectures and loss functions of PS-Net on U-RISC dataset.

Method	$L_{1}$	$L_{2}$	$L_{3}$	*F*1 (%)	V-Rand (%)	V-Info (%)	PHD-0↓	PHD-5↓	PHD-10↓	PHD-50↓
$S_{G}$	✓			51.57	53.01	53.92	21.61	19.59	10.42	7.227
$S_{L}$	✓			58.23	56.94	57.05	23.53	20.41	12.92	8.039
$S_{G}+S_{L}$	✓			59.57	58.71	59.80	20.91	17.04	9.367	6.294
$S_{G}$	✓	✓		53.81	54.78	54.79	17.58	16.11	8.829	3.142
$S_{L}$	✓	✓		61.98	63.62	61.03	17.14	16.32	8.994	3.035
$S_{G}+S_{L}$	✓	✓		66.20	67.24	65.00	16.07	15.21	7.878	2.770
$S_{G}+S_{L}$	✓	✓	✓	**67.69**	**68.93**	**65.32**	**15.29**	**13.52**	**6.969**	**1.594**

The boldface values indicate the best performance.

#### 6.2.2 Global–local strategy

To investigate the effectiveness of the global–local strategy, we conducted experiments using three architectures: $S_{G}$, $S_{L}$, and $S_{G}$+$S_{L}$. Results presented in Table 3 indicate that the combined approach of $S_{G}$+$S_{L}$ outperforms either $S_{G}$ or $S_{L}$ alone. When using only pixel-wise loss, the *F*1 score of $S_{G}$+$S_{L}$ is 59.57% compared to 51.57% for $S_{G}$ and 58.23% for $S_{L}$. Similar improvements were observed on other evaluation criteria. These results suggest that the global–local strategy can be advantageous in segmentation, as it not only increases the local accuracy but also alleviates the global structure distance. Moreover, to provide further insight into the impact of PS-Net, we have illustrated the feature visualization in Supplementary Fig. S6 and conducted an attribution analysis for the global–local strategy in Supplementary Fig. S9. Our results indicate that the model trained with this strategy is able to capture more structural information, with a larger number of pixels contributing significantly to the prediction. These findings suggest that the global–local strategy enables the network to effectively utilize features of larger regions, thereby improving the segmentation performance. Due to space limitations, we have provided additional information in the Supplementary Materials.

#### 6.2.3 Coarse-to-fine strategy

Additionally, the experiments were conducted to explore the effectiveness of the coarse-to-fine strategy by varying the parameters $\lambda_{1}$, $\lambda_{2}$, and *k*, as presented in Table 3 and Supplementary Table S5. The results indicate that gradually increasing the weights of the similarity loss and PHD loss resulted in improved segmentation performance. Notably, when the epoch is set to five, the introductions of the similarity loss and PHD loss yielded the best performance. These findings suggest that the coarse-to-fine strategy, with appropriate parameter tuning, can effectively improve the accuracy of segmentation tasks.

### 6.3 Experiments on natural image datasets

We further extend PS-Net to two natural image datasets: “Road” (Mnih 2013) and “CrackTree” (Zou *et al.* 2012). For evaluation, Pixel-wise accuracy, ARI, VOI, and Betti are chosen for comparison [reported by (Hu *et al.* 2021)]. The results in Table 4 also show that our work has SOTA performance. It is worth mentioning that PS-Net obtained a much better VOI score (**0.5117**) on the Road dataset.

**Table 4.** Quantitative results on Road, and CrackTree datasets.

	Road			
Methods	Acc	ARI	VOI↓	Betti↓
DIVE	0.9734±0.01	0.8201±0.01	2.368±0.20	3.598±0.78
U-Net	**0.9786** ± **0.01**	0.8189±0.01	2.249±0.18	3.439±0.62
Mosin.	0.9754±0.01	0.8456±0.02	1.457±0.10	2.781±0.24
TopoLoss	0.9728±0.01	0.8671±0.01	1.234±0.04	1.275±0.19
DMT	0.9744±0.01	0.8819±0.01	1.092±0.13	0.995±0.30
PS-Net	0.9785±0.01	**0.8811** ± **0.01**	**0.5117** ± **0.09**	**0.898** ± **0.19**

	CrackTree			
Methods	Acc	ARI	VOI↓	Betti↓
DIVE	0.9854±0.01	0.8634±0.04	1.570±0.08	1.576±0.29
U-Net	0.9821±0.01	0.8749±0.04	1.625±0.10	1.785±0.30
Mosin.	0.9833±0.01	0.8897±0.02	1.113±0.06	1.045±0.21
TopoLoss	0.9826±0.01	0.9291±0.01	0.997±0.01	0.672±0.18
DMT	0.9842±0.01	**0.9307** ± **0.02**	0.901±0.08	0.518±0.19
PS-Net	**0.9957** ± **0.01**	0.9038±0.02	**0.901** ± **0.02**	**0.512** ± **0.15**

The boldface values indicate the best performance.

## 7 Conclusions

In this study, we propose a novel criterion PHD and a PHD-based network for the task of cell membrane segmentation in EM images. The motivation for this approach arose from the discrepancy between commonly used metrics and human evaluations of segmentation results. To gain insight into the way humans analyze differences between segmentations, we conducted eye movement tracking experiments. These experiments revealed that humans utilize “global-local” and “coarse-to-fine” strategies in this process. Based on these observations, we incorporated these strategies into our model through the use of separate global and local networks and the inclusion of PHD-based losses after initializing training with pixel-wise loss. Our proposed method was evaluated on several public EM and natural image datasets with consistently high performance.

## Supplementary Material

btad464_Supplementary_Data

## Data Availability

The data underlying this article is available in https://github.com/EmmaSRH/PS-Net.
