# Supplementary material for: PS-Net: human perception-guided segmentation network for EM cell membrane
Source: Bioinformatics. 2023 Jul 28;39(8):btad464. doi: 10.1093/bioinformatics/btad464 (PMC10423022; doi:10.1093/bioinformatics/btad464)
Supplement: btad464_Supplementary_Data [file btad464_supplementary_data.pdf]

Supplementary of
PS-Net: Human perception-guided segmentation network for EM cell membrane

Ruohua Shi[1,2], Keyan Bi[3], Kai Du[4], Lei Ma[1,2], Fang Fang[3],
Lingyu Duan[1], and Tingting Jiang[1,*]

[1]National Engineering Research Center of Visual Technology, School of Computer Science, Peking University, China
[2]Beijing Academy of Artificial Intelligence, Beijing, China
[3]IDG/McGovern Institute for Brain Research, School of Psychological and Cognitive Sciences, Peking University, China
[4]Institute for Artificial Intelligence, Peking University, China
[*]Corresponding author

May 19, 2023

1 Perceptual consistency experiments.

1.1 Interface of perceptual consistency experiments.

Supplementary Fig. 1 shows the interface of perceptual consistency experiments.

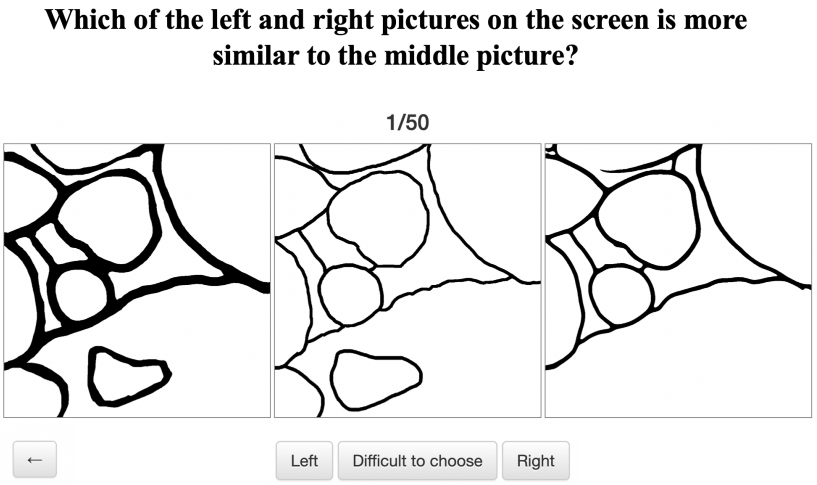

Figure 1: Interface of subjective experiments. The three images form a group. Middle: manual annotation (ground truth) of an EM image. Two sides: predictions of the same EM image.

1.2 The details of subjective experiments design.

In this section, we provide more details for the subjective experiment (in Sec. 3 of the manuscript).

Before the experiment, the 20 subjects were introduced the value of cell membrane segmentation to connectivity and the importance of structure before testing. And then 10 simple examples are used to teach them about the experimental process.

The 200 groups of images for the subjective experiment were randomly selected from the segmentation results produced by six segmentation methods (U-Net, SENet, LinkNet, GLNet, CASENet, and U-Net++). And the distribution and selection of data during the experiment were random.

During the experiment, the 200 groups of images were equally divided into four batches to prevent subjects from experiencing fatigue during evaluation. And the subjects were instructed to select one of

two images that they believed most closely resembled the ground truth (left or right). If the subject was uncertain, they could select “Difficult to choose”. Continuous judgment without interruption was required for each batch of images.

After the experiment, three concepts were defined to calculate the consistency of a metric with human judgment:

- “valid group”: for each group of images, if there were more than a half (10) of the subjects choose the same segmentation (left of right), then, this group was called a “valid group”.
- “human choice”: for each ”valid group”, the image chosen by most people was considered the ”human choice”.
- “metric choice”: for each ”valid group”, the segmentation, whose score calculated by the metric shows that it is more similar to the ground truth, was considered the ”metric choice”.

A total of 113 valid groups were identified, and the consistency was defined as N(human choice = metric choice)/N(valid groups).

To maintain continuity, participants were required to complete the judgment of each group within a specified time (less than 10 minutes) and complete each batch’s judgment continuously without interruption.

1.3 More examples of subjective experiment cases.

In the subjective experiments, more examples of images are shown in Supplementary Fig. 2.

2 Eye movement Experiments.

We use the EyeLink 1000 Plus to record the saccades, which has built-in saccade detection algorithms that can be used to detect saccades automatically. First, we calibrate the eye tracker according to the manufacturer’s instructions to ensure accurate tracking of eye movements. Then, a stimulus is set up to display that will be presented to the participant. Next, we show two images like Supplementary Fig. 3 on the stimulus display for subjects. The EyeLink 1000 Plus will record eye movements as the subject looks at the stimulus display. We use EyeLink’s software development kit (SDK) to write code that will detect saccades based on the eye movement data recorded during the experiment. Finally, we analyze the eye movement data by the fixation and saccades.

2.1 Interface of eye movement experiments

Supplementary Fig. 3 shows the interface of perceptual consistency experiments.

2.2 Fixation maps of eye movement experiments

Supplementary Fig. 4 shows some examples of average fixation map from 20 subjects in the eye movement experiments.

2.3 Saccades of eye movement experiments

Supplementary Fig. 5 shows some examples of saccades from 20 subjects in the eye movement experiments.

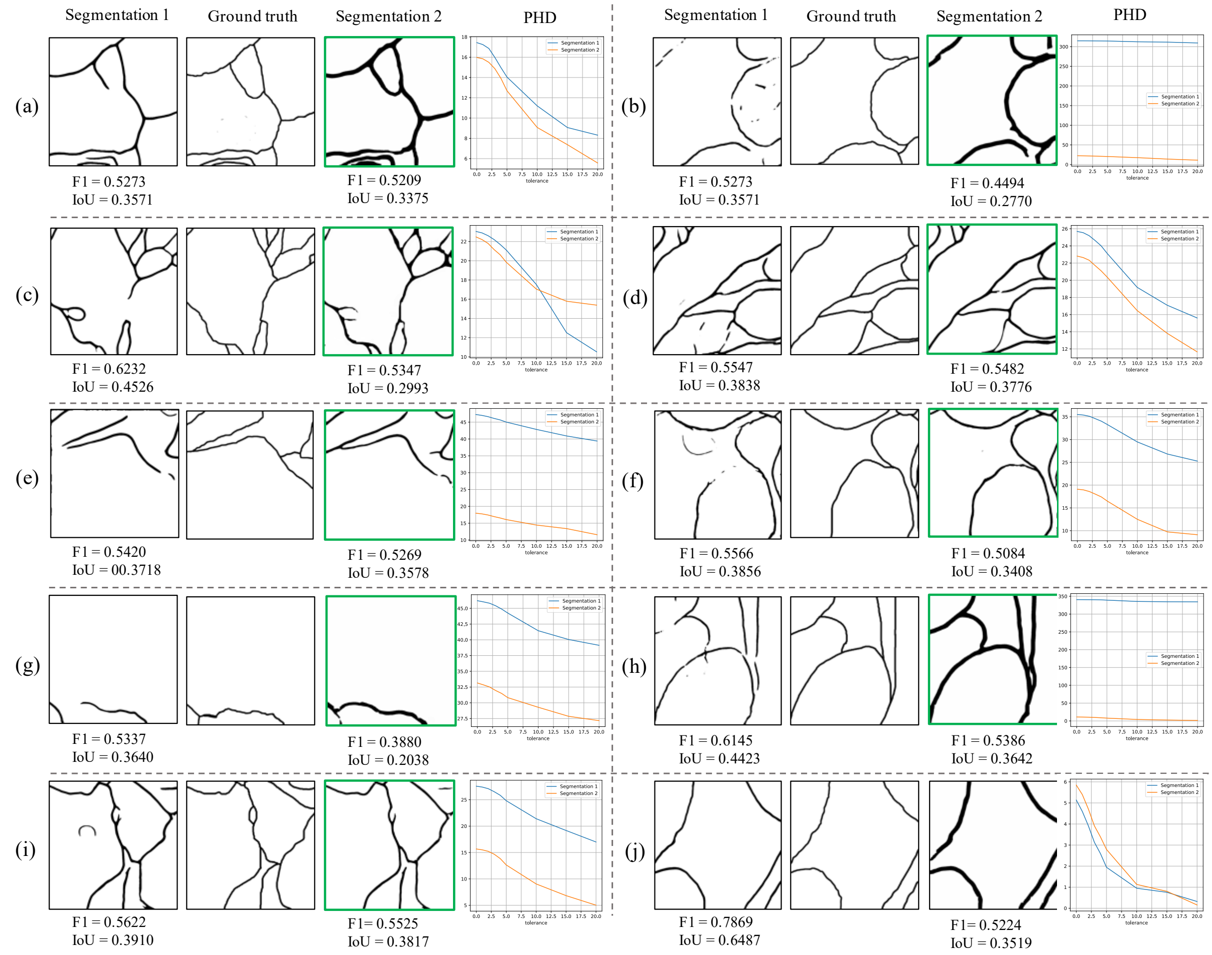

Figure 2: Examples of subjective experiment images. Each row shows two groups of images with their scores. For each group (a)-(j), The four images from left to right are: the segmentation of one method, the ground truth image, the segmentation of the other method, and PHD scores with different tolerance thresholds of the two segmentations. In each group, the figure with green box is the choice of most subjects. The two scores (F1 and IoU) below the segmentation results are the evaluation of the segmentation based on the ground truth, which are not shown to the subjects during the subjective experiments. For (j) which has no green box in the last row, it means that most of the subjects chose "Difficult to choose". The horizontal axis of the PHD scores represents the tolerance threshold.

3 Formulas of evaluation metrics.

Supplementary Tab. 1 shows the formulas of evaluation metrics used in the manuscript.

- TP (true positives), TN (true negatives), FP (false positives), and FN (false negatives) compare the segmentation results with ground truth. The terms positive and negative refer to the classifier's prediction, and the terms true and false refer to whether that prediction corresponds to the ground truth.
- X and Y are two point sets. x and y are the points in X and Y respectively.
- For V-Rand, suppose that S is the predicted segmentation and W is the ground truth segmentation. Define p_{ij} as the probability that a randomly chosen pixel belongs to segment i in S and segment j in W. This joint probability distribution satisfies the normalization condition $\sum_{ij} p_{ij} = 1$. The marginal distribution $s_i = \sum_j p_{ij}$ is the probability that a randomly chosen pixel belongs to segment i in S, and the marginal distribution $w_j = \sum_i p_{ij}$ is defined similarly. For V-Info, the mutual information $I(S;W) = \sum_{ij} p_{ij} \log p_{ij} - \sum_i s_i \log s_i - \sum_j w_j \log w_j$ is a measure of similarity between S and W. $H(S) = -\sum_i s_i \log s_i$ is the entropy function. α is a manually

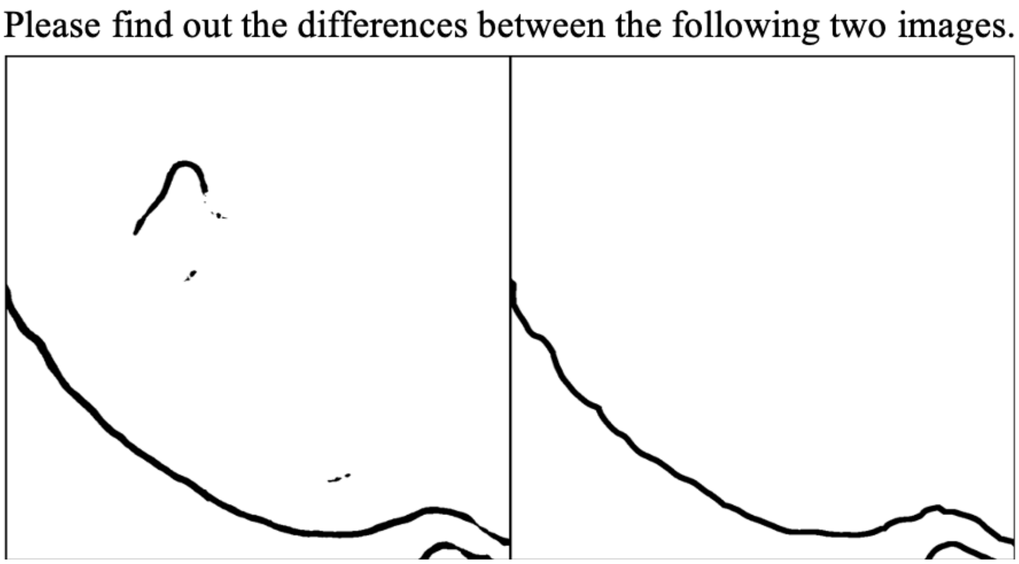

Figure 3: Interface of eye movement experiments. The two images form a group.

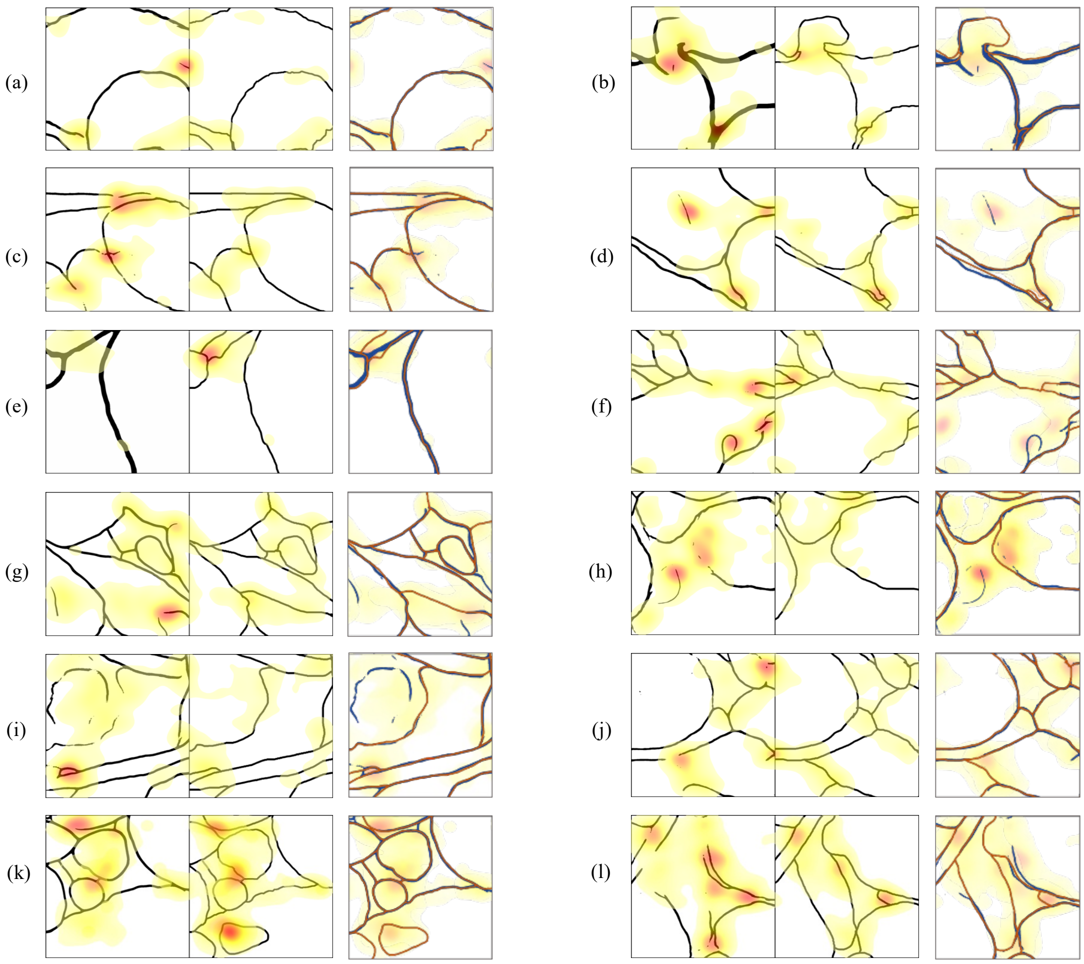

Figure 4: Examples of average fixation maps. In each column, **(left):** the overlap of fixation map with the two images: prediction and ground truth. The heatmaps show the accumulated time of fixations (red: long, yellow: middle, white: short). **(right):** the overlap of two images and two fixation maps of the left image.

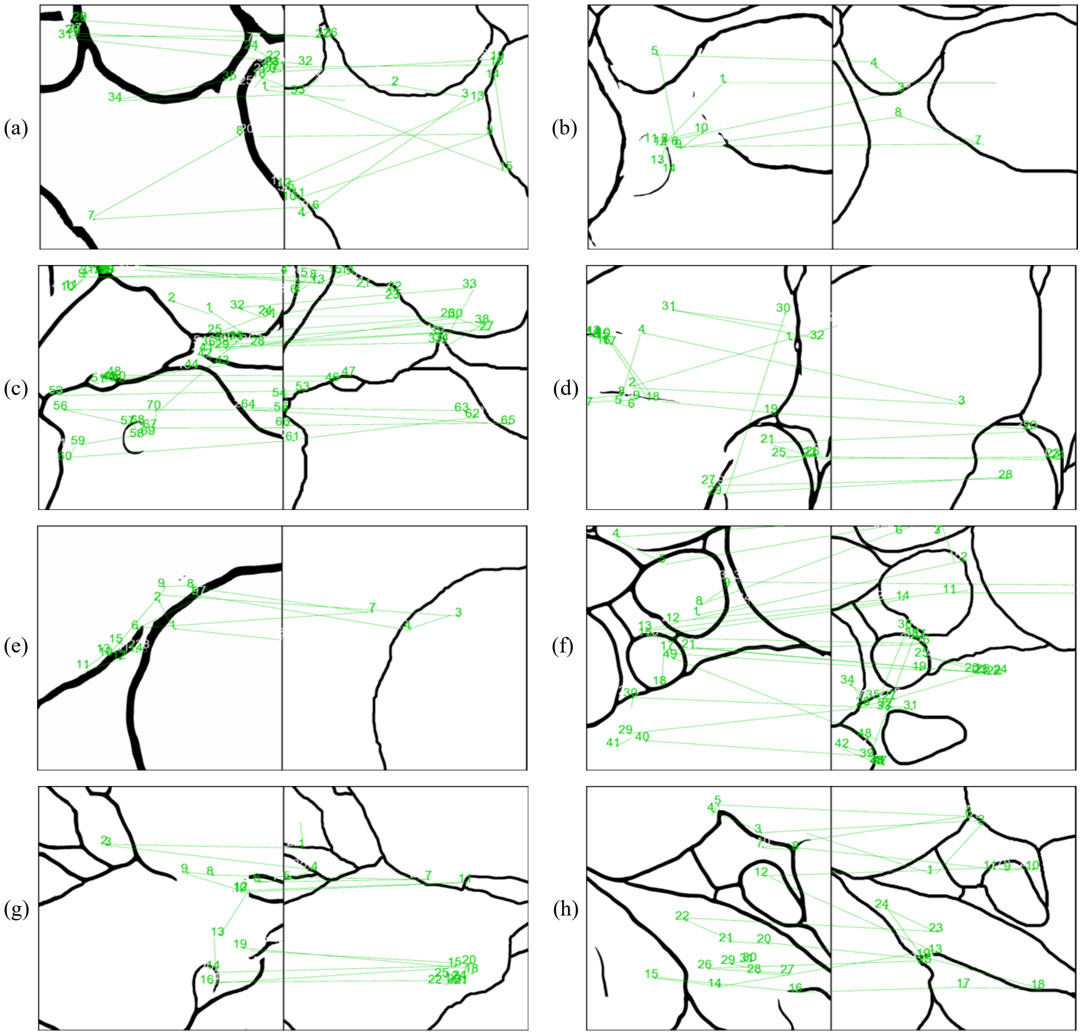

Figure 5: Examples of saccades. The green arrows represents the directions of saccades. The number means the order.

Table 1: Formulas of criteria.

**Criteria**	**Formulation**
Precision	$\frac{TP}{TP+FP}$
F1 score	$\frac{2\times precision\times recall}{precision+recall}$
Dice	$\frac{2\times TP}{2\times TP+FP+FN}$
IoU	$\frac{TP}{TP+FP+FN}$
TPVF	$\frac{TP}{TP+FN}$
TNVF	$\frac{TN}{FP+TN}$
RVD	$\left\lvert\frac{FP-FN}{TP+FN}\right\rvert$
RI	$\frac{TP+TN}{TP+FP+TN+FN}$
ARI	$\frac{RI-E(RI)}{max(RI)-E(RI)}$
MI	$H(S)+H(W)-H(S,W)$
VOI	$H(S)+H(W)-2MI(S,W)$
Hausdorff	$\max\left\{\max_{x\in X}\left\{\min_{y\in Y} d(x,y)\right\},\max_{y\in Y}\left\{\min_{x\in X} d(x,y)\right\}\right\}$
ASSD	$\frac{1}{\lvert X\rvert+\lvert Y\rvert}\left(\sum_{x\in X}\min_{y\in Y} d(x,y)+\sum_{y\in Y}\min_{x\in X} d(x,y)\right)$
V-Rand	$V_{\alpha}^{\text{Rand}}=\frac{\sum_{ij} p_{ij}^2}{\alpha\sum_k s_k^2+(1-\alpha)\sum_k w_k^2}$
V-Info	$V_{\alpha}^{\text{info}}=\frac{I(S;W)}{(1-\alpha)H(S)+\alpha H(W)}$
clDice	$clDice(V_P,V_L)=2\times\frac{\text{Tprec}(S_P,V_L)\times\text{Tsens}(S_L,V_P)}{\text{Tprec}(S_P,V_L)+\text{Tsens}(S_L,V_P)}$<br>$\text{Tprec}\left(S_P,V_L\right)=\frac{\lvert S_P\cap V_L\rvert}{\lvert S_P\rvert};\quad\text{Tsens}\left(S_L,V_P\right)=\frac{\lvert S_L\cap V_P\rvert}{\lvert S_L\rvert}$
PHD	$\frac{1}{\lvert X\rvert}\sum_{x\in X}\min_{y\in Y}\Psi(x,y)+\frac{1}{\lvert Y\rvert}\sum_{y\in Y}\min_{x\in X}\Psi(x,y)$<br>$\Psi(x,y)=\begin{cases}\lVert x-y\rVert, & \lVert x-y\rVert>\tau\\ 0, & \lVert x-y\rVert\leq\tau\end{cases}$

set parameter, and usually it is 0.5. For the implementation code for V-Rand and V-Info, see https://imagej.net/tutorials/segmentation-evaluation-after-border-thinning.

- Betti error: which directly compares the topology (number of handles) between the segmentation and the ground truth. We randomly sample patches over the segmentation and report the average absolute difference between their Betti numbers and the corresponding ground truth patches.
- In PHD, the τ represents the tolerance threshold.

4 More details of segmentation experiments.

4.1 Datasets

ISBI 2012 dataset (1) contains the Drosophila ventral nerve cord serial-section electron microscopy data, which was captured from a first instar larva. For training, the provided set included a 2×2×1.5 μm^3 volume imaged from 30 sections and publicly available manual segmentations. For testing, the provided set included only image data, with segmentations kept private for the assessment of segmentation accuracy. The size of each image is 512 × 512.

U-RISC dataset (2) was annotated upon RC1, a large-scale retinal serial section transmission electron microscopic dataset. RC1 came from the retina of a light-adapted female Dutch Belted rabbit after in vivo excitation mapping. The imaged volume represents the retinal tissue with a diameter of 0.25 mm, spanning the inner nuclear, inner plexiform, and ganglion cell layers. Serial EM sections were cut at 70-90 nm with a Leica UC6 ultramicrotome and captured at the resolution of 2.18 nm/pixel across both axes using SerialEM. There are two tracks in the public challenge of U-RISC, and we choose the track with higher resolution of images in our experiments. For training and testing, the provided set included a total of 50 and 20 images sections and publicly available manual segmentations. The size of each image is 9989 × 9959.

Road dataset (3) has 1108 images from the Massachusetts Roads Dataset, which is one of the largest publicly available collections of aerial road images, containing both urban and rural neighborhoods, with many different kinds of roads ranging from small paths to highways. The resolution for each image is 1500×1500. In our experiments, the set is split into 1108 training and 49 test images.

CrackTree dataset (4) contains 206 images of cracks in the road. The resolution for each image is 600×800. The multiple shadows and cluttered background makes their detection a challenging task. Potential applications include quality inspection and material characterization.

4.2 Baseline methods.

In this paper, we use eight baseline methods for the segmentation of U-RISC datasets as follows:

U-Net (5) is a fully convolutional neural network designed for image segmentation tasks. The U-Net architecture consists of an encoder and a decoder, which are connected by a series of skip connections. The encoder consists of a series of convolutional and pooling layers, which extract high-level features from the input image. The decoder then uses up-sampling and concatenation operations to recover the spatial resolution of the image and produce the final segmentation mask.

CASENet (6) is designed for semantic segmentation. The architecture consists of three modules: feature extraction, context modeling, and semantic segmentation. The feature extraction module extracts features from the input image, while the context modeling module captures contextual information at multiple scales using dilated convolutional layers. The semantic segmentation module then produces the final segmentation mask using convolutional layers.

LinkNet (7) is based on a modified U-Net architecture with a series of skip connections. The network consists of an encoder and decoder connected by a series of residual connections. The encoder consists of a series of convolutional layers followed by max pooling, while the decoder consists of a series of up-sampling and convolutional layers. The skip connections are added between the corresponding layers in the encoder and decoder, allowing the network to effectively use both low-level and high-level features for segmentation.

GLNet (8) is designed for semantic segmentation of high-resolution images. The network consists of three modules: global feature extraction, local feature extraction, and feature fusion. The global feature extraction module captures the global context information of the input image using a series of convolutional layers. The local feature extraction module extracts detailed local features using dilated convolutional layers. The feature fusion module combines the global and local features using a fusion block, which consists of convolutional and pooling layers.

SENet (9), or Squeeze-and-Excitation Network, is a deep neural network architecture designed to improve the performance of convolutional neural networks (CNNs) by explicitly modeling the interdependencies between channels. The architecture consists of a series of convolutional layers followed by a squeeze-and-excitation (SE) block. The SE block consists of a squeeze operation, which reduces the spatial dimensions of the input feature maps to a single value, followed by an excitation operation, which applies a non-linear transformation to the squeeze output.

U-Net++ (10) is a deep neural network architecture designed for semantic segmentation of images. The architecture was proposed in 2018 and is based on the popular U-Net architecture, but with a series of modifications to improve performance. The network consists of an encoder and decoder, similar to the

Table 2: IoU results of the methods on ISBI 2012 dataset. The upper results are tested in this work, and the lower results are reported in (14).

Method	IoU(%) ± Std
U-Net(5)	92.31 ±0.0141
CASENet(6)	89.61±0.0032
LinkNet(7)	91.02±0.0021
GLNet(8)	81.89±0.0172
SENet(9)	84.24±0.0073
U-Net++(10)	89.56±0.0165
Inception v3(15)	88.63±0.0074
MultiResUNet(16)	91.96±0.0121
EfficientNet-b0(17)	88.48±0.0051
DC-Unet(18)	92.62±0.0092
MobileNet v2(19)	89.06±0.0011
ICNet(20)	69.67±0.0189
ESPNet(21)	84.29±0.0179
ENet(22)	67.34±0.0276
CFPNet-M(14)	91.44±0.0111
PS-Net	**93.99±0.0015**

U-Net architecture, but with a series of nested dense skip connections added between the corresponding layers in the encoder and decoder.

Mosin. (11) builds on the popular U-Net architecture, but with modifications to incorporate topological constraints. The network consists of an encoder and decoder, similar to the U-Net architecture, but with the addition of a topological loss function. The topological loss function penalizes deviations from the expected topology of the segmented structures, such as disconnected regions or spurious holes.

DMT (12) represents the topological structure of the image using a set of critical points and their connecting paths. The Morse complex is then used to guide the segmentation process, by identifying regions of interest and constraining the segmentation to follow the topological structure of the image.

The eight methods employ identical training and testing data sets for performance comparison, utilizing parameters and loss functions proposed in their respective references.

4.3 Experiment settings

The proposed deep network is implemented using PyTorch open-source deep learning library (13). Training and deployment of the network are conducted on an NVIDIA GTX Geforce A100 GPU. The data augmentation method is used for the input images by applying rotation and shift transformations for training. The number of patches N is set to 4. The tolerance threshold τ in loss functions is set to 2. The weights λ_1 and λ_2 of the final loss function are both set to 0 at the first k epochs. From the $k+1$ epoch, as the number of training epoch increases, the weight also increases by 0.1 for every 10 epochs until it reaches 1. The k here is set to 5. The increase ratio in each epoch is the variance of L_{phd} and L_{sim} of the last two epochs, normalized by the number of skeleton points. For all datasets, we use a three-fold cross-validation.

Because these methods for comparison do not provide the predictions and the results of other evaluation metrics, the results of Tab. 1 and Tab. 2 in the manuscript are obtained by training and testing with the official code. The results of Tab. 3 are reported by the original articles. For the typesetting of the article, we report them separately.

4.4 More IoU results on ISBI 2012 dataset.

Supplementary Tab. 2 shows more IoU results on ISBI 2012 dataset.

4.5 Comparison on the leaderboards.

Supplementary Tab. 3 and Supplementary Tab. 4 show the comparisons of PS-Net on the leaderboards of ISBI 2012 and U-RISC. The results of comparison methods are reported by the original articles.

Table 3: V-Rand and V-Info results of the methods on ISBI 2012 testing dataset. For full ranking of all submitted methods, please refer to the challenge website. The comparison scores are reported by the original papers.

Method	V-Rand(%)	V-Info(%)
U-Net(5)	94.32	95.62
CE-Net(23)	97.43	98.78
M2FCN(24)	97.80	-
FusionNet(25)	97.80	98.99
IAL IC(26)	97.73	98.92
CUMedVision(27)	97.68	98.86
TAD(11)	98.1	-
PatchPerPix(28)	**98.83**	99.15
MWS(29)	0.9879	**99.18**
**PS-Net**	98.73	99.09

Table 4: F1 scores of the methods on U-RISC dataset. See the challenge website for full ranking of all submitted methods.

Team	VIDAR	SpongeBobbb	yangsenwxy	SCP	PS-Net
F1(%)	60.30	60.48	60.70	60.70	**67.69**

4.6 Ablation study for the tolerance distance.

For the function f^+ and f^-, there are varies of optional formulas. Here we choose the formula as sigmoid-like, tanh-like, and ReLU-like for comparision. The difference between sigmoid-like and sigmoid is the inflection point, where it is 0 in the sigmoid, while the tolerance threshold τ in the sigmoid-like, and others are the same. In our experiments, the three formula have the same consistency with human beings, while the computational cost of ReLU-like is minimal. So we choose $f^+(d) = d$ and $f^-(d) = 0$.

When selecting the tolerance for the PHD loss function during the training phase, multiple factors should be considered, including the size of the dataset, and the desired level of accuracy. Generally, lower tolerance values can increase precision but also raise the risk of overfitting, while higher tolerance values can result in a more generalized model but potentially compromise accuracy.

To investigate the optimal tolerance for the PHD loss function, ablation experiments were conducted by varying the tolerance and observing its effect on the model's performance. This provides insights into the trade-offs between precision and generalization and helps identify the ideal tolerance for the specific task and dataset at hand. The results of the ablation experiments in Supplementary Tab. 5 demonstrate that the proposed PS-Net attains superior performance when τ equals 2, as evidenced by the scores of 15.29/13.52/6.979 (PHD-0/5/10). We also observed that increasing or decreasing the tolerance led to a decrease in performance. Therefore, we chose $\tau = 2$ for subsequent experiments.

4.7 Feature visualization for PHD loss function on U-RISC dataset

Supplementary Fig. 6 shows the feature visualization for PHD loss function on U-RISC dataset.

4.8 More results of cell membrane segmentation on ISBI 2012 dataset.

More examples for the segmentation results of ISBI 2012 dataset (in Sec. 5.3 of the submission) are shown in Supplementary Fig. 7.

4.9 More results of cell membrane segmentation on U-RISC dataset.

More examples for the segmentation results of U-RISC dataset (in Sec. 5.4 of the submission) are shown in Supplementary Fig. 8.

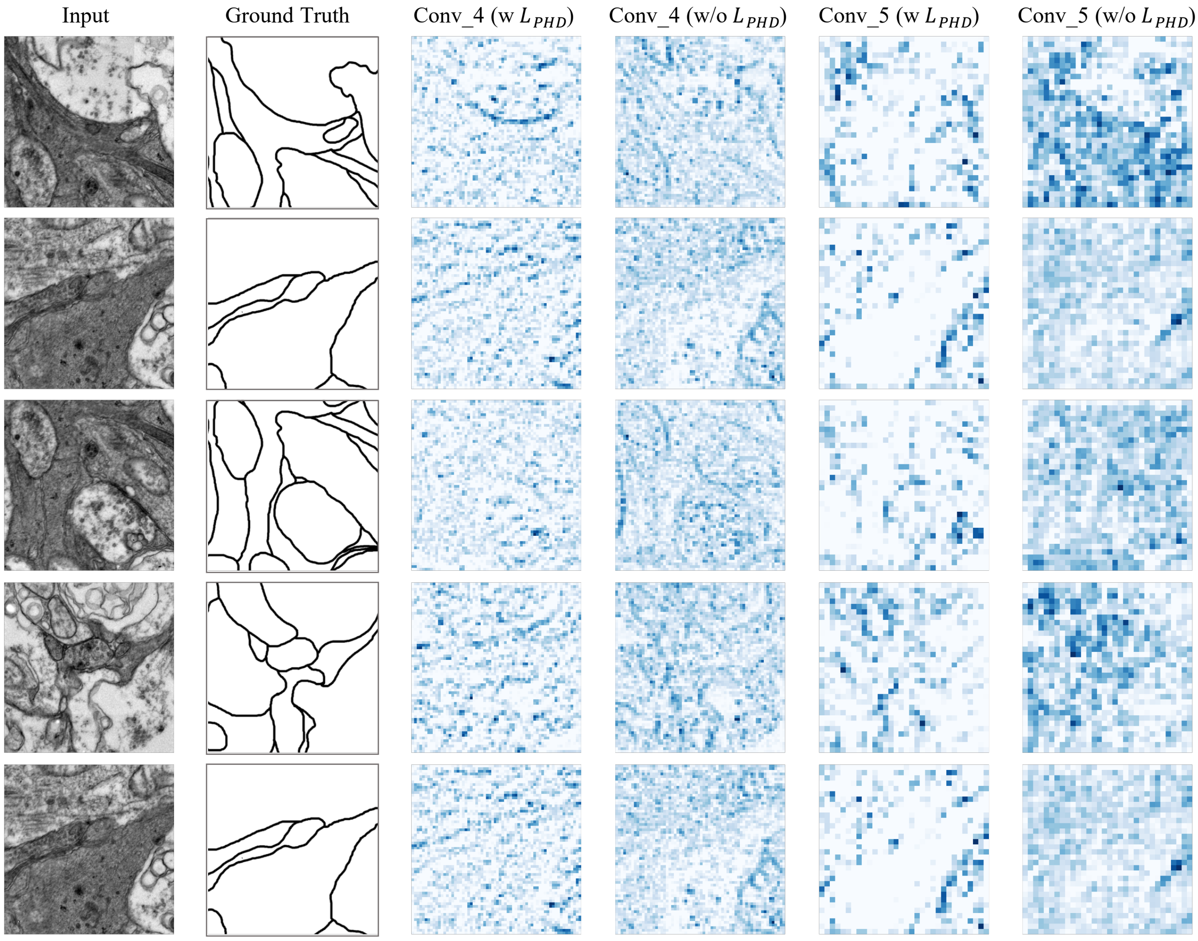

Figure 6: Feature visualization of U-Net on U-RISC dataset.

4.10 Ablation studies for the hyperparameters on U-RISC dataset.

In our experiments, the values for λ_1 and λ_2 are first set to 0 at the first k epochs, and as the number of training epoch increases, the weight also increases by 0.1 for every 20 epochs until it reaches 1. In this section, we test the fixed $lambda_1$ and $lambda_2$ including (0.5, 0.5), (1,0), (0,1), and (1, 1), and we found that the best combination was the adaptive one, which produced the highest accuracy metrics. The results are shown in the Supplementary Tab. 5.

We also tested different values for k, including 0, 3, 5, 10, and 15. We found that increasing k beyond 5 did not significantly improve the results. A small k can also degrade performance with more computational costs. Therefore, we used a value of $k = 5$ for our experiments, which struck a balance between accuracy and efficiency.

We acknowledge that there may be other combinations that could also produce good results. Therefore, we suggest that future research could explore different combinations of lambda1, lambda2, and k to further understand the impact of these parameters.

4.11 Ablation studies for the topology loss.

Further, we also compared the PHD loss with another topology loss, clDice loss (30), using the same network architecture as PS-Net on the U-RISC dataset. In PS-Net, the PHD loss is used for two purposes: firstly, it is used to measure the dissimilarity between the ground truth and the predictions by the global and local branches respectively, and secondly, it is used to measure the dissimilarity between the predictions of the two branches. In the new experiments, we replaced both of them with the clDice loss, and kept all other parameters the same as those used in Table 2 of the manuscript. The equation of the clDice loss is:

$$L = (1 - \alpha)(1 - \text{ softDice }) + \alpha(1 - \text{ softclDice }) \tag{1}$$

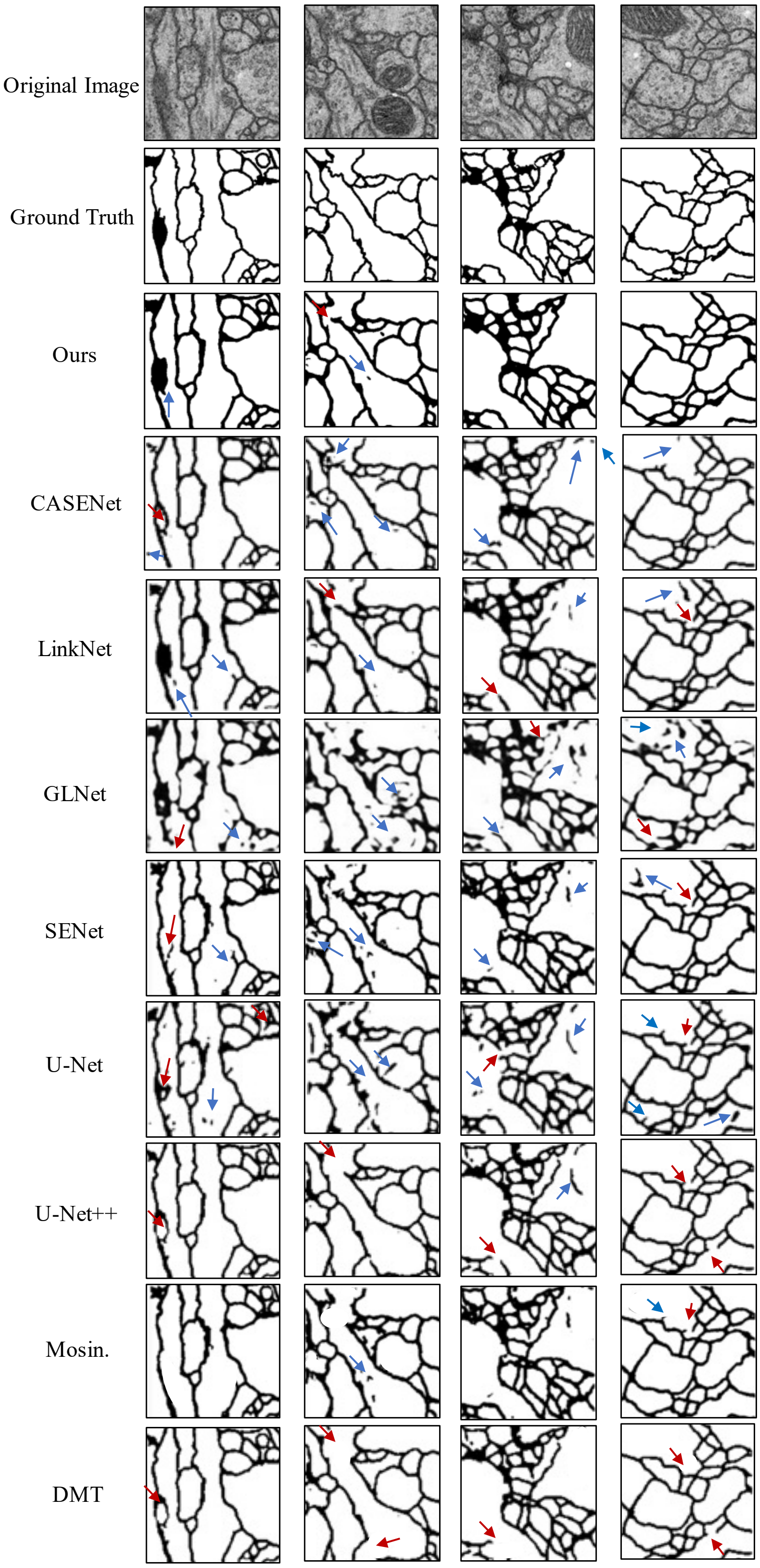

Figure 7: Segmentation results of ISBI 2012 dataset. Red arrow: false negative error. Blue arrow: false positive.

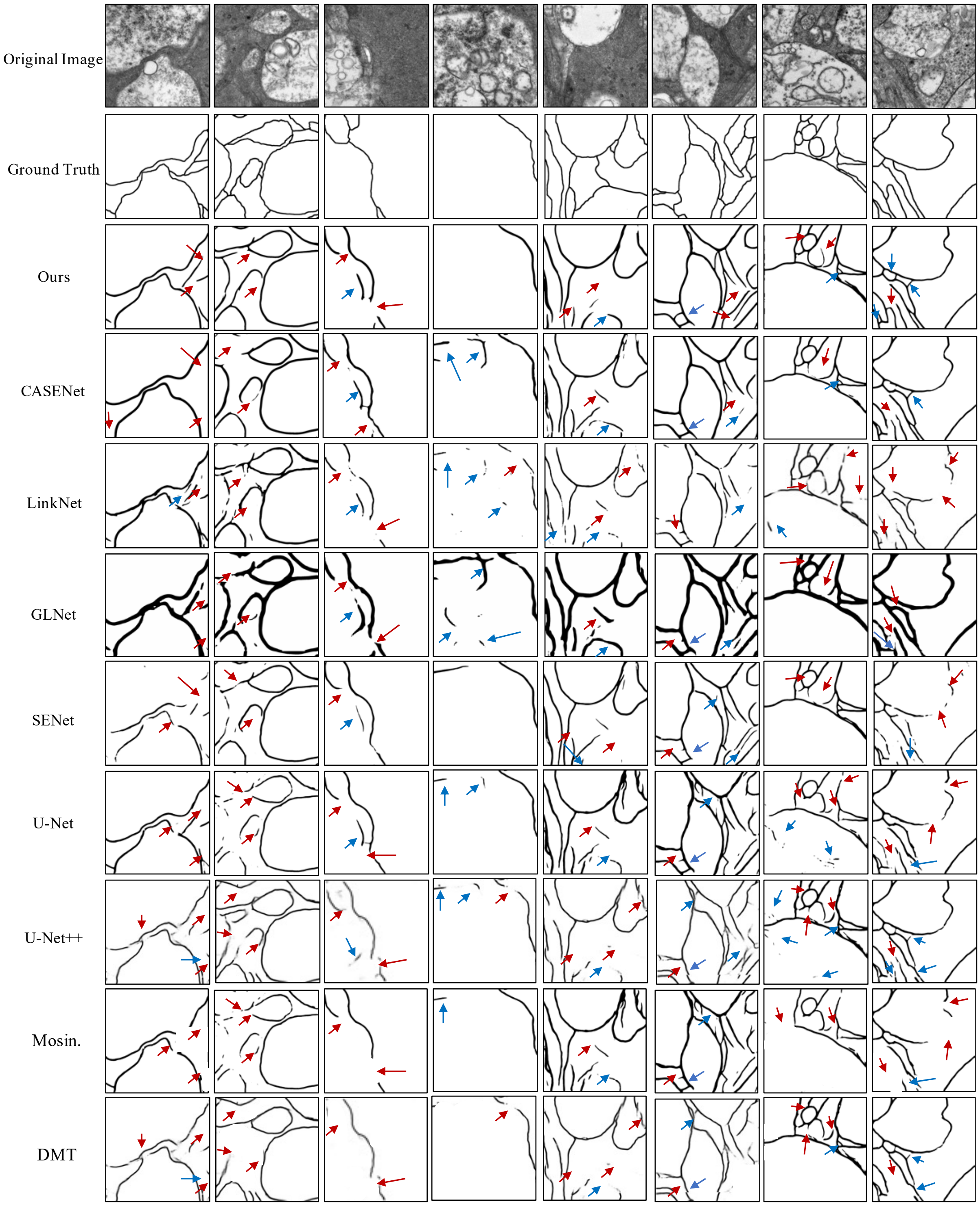

Figure 8: Segmentation results on U-RISC dataset. Red arrow: false negative error. Blue arrow:false positive.

Table 5: Ablation studies for the hyperparameters on U-RISC dataset.

(λ_1, λ_2)	(0.5, 0.5)	(1,0)	(0,1)	(1, 1)	original
PHD-0	16.30	17.99	16.73	19.79	**15.29**
PHD-5	14.06	14.40	15.52	15.88	**13.52**
k	0	3	5	10	15
PHD-0	20.98	15.03	**15.29**	15.56	15.62
PHD-5	15.24	14.11	**13.52**	13.71	13.84
tau	0	1	2	4	8
PHD-0	18.32	15.97	**15.29**	15.36	17.92
PHD-5	15.44	13.78	**13.52**	13.89	16.01
PHD-10	8.361	7.255	**6.979**	7.196	8.640

where α is the parameter in the clDice loss. In accordance with the experimental setup of clDice loss (30), we trained the network by varying the value of α from 0.1 to 0.5. Our results in Supplementary Tab. 6 indicate that the utilization of PHD loss leadsOur results in Supplementary Tab. 6 indicate that the utilization of PHD loss leads to higher scores compared to clDice loss across all α values, highlighting the superiority of PHD loss in effectively segmenting cell membranes in EM images. Furthermore, when specifically considering the performance of the clDice loss, the model consistently achieved the highest performance for the PHD, F1, V-Rand and V-Info metrics at $\alpha = 0.4$, (with the best IoU at $\alpha = 0.5$),while for the F1 metric, the best performance was observed at $\alpha = 0.1$.

Table 6: Ablation studies for the topology loss.

Metrics		F1↑	clDice↑	IoU↑	V-Rand↑	V-Info↑	PHD-0↓	PHD-3↓	PHD-5↓	PHD-10↓	PHD-50↓
clDice loss	$\alpha = 0.1$	<u>63.87</u>	67.94	39.91	66.01	61.39	17.11	16.90	15.22	8.41	4.87
	$\alpha = 0.2$	63.59	68.26	39.99	66.33	61.58	17.24	16.97	15.21	8.52	4.91
	$\alpha = 0.3$	63.62	68.08	40.15	66.34	61.80	17.37	17.14	15.10	8.48	4.85
	$\alpha = 0.4$	63.76	<u>68.21</u>	40.22	<u>66.56</u>	<u>62.15</u>	<u>16.74</u>	<u>16.86</u>	<u>15.09</u>	<u>8.31</u>	<u>4.79</u>
	$\alpha = 0.5$	63.58	68.20	<u>40.23</u>	66.50	62.14	17.01	16.87	15.17	8.36	4.80
PHD loss		**67.69**	**70.45**	**43.63**	**68.93**	**65.32**	**15.29**	**15.01**	**13.52**	**6.98**	**1.59**

5 Complexity of Methods.

To analyze the running efficiency of our PS-Net, we compare PS-Net with other six networks: U-Net, SENet, LinkNet, GLNet, CASENet, and U-Net++. The performance of two measurements are reported: the number of parameters and the floating-point operations (FLOPs). The experiments are performed on a single Nvidia A100 GPU. And for a quick testing, the resolution of input images is chosen as 512×512 for all the methods. As shown in Supplementary Tab. 7, we observe that the number of parameters and FLOPs of our method are similar to U-Net, but smaller than GLNet. The main reason is that we use U-Net as default segmentation module, and the global-local strategy does not add more parameters in the network. Besides, the local branch of PS-Net uses the input with 1/4 size of the original image, resulting in a geometrically less FLOPs compared with the original size of the image. With the acceptable computational complexity similar to that of U-Net, our method has the best performance (F1 score and PHD).

Table 7: Complexity and performance of methods.

**Model**	**Paremeters(M)**	**FLOPs(G)**	**F1 score(%)**	**PHD-0**$\downarrow$
U-Net	34.53	261.82	48.83	18.65
SENet	47.25	41.05	52.12	20.42
GLNet	158.22	429.51	58.10	23.30
CASENet	42.43	206.58	60.07	19.25
U-Net++	41.85	225.17	60.30	17.25
LinkNet	**11.54**	**12.74**	60.70	22.72
PS-Net	34.53	327.27	**67.69**	**15.29**

6 Attribution analysis for global-local strategy.

To acquire a deeper understanding of the impact of the global-local strategy on the network, we added an attribution analysis on the trained models with/without global-local strategy by a gradient-based attribution method, integral gradient (31). In brief, IG aims to explain the relationship between the predictions and the input image or features based on gradients. To be specific, we choose one of pixels in the segmentation result, and then explore which areas of the input image contribute more to its decision. The output is a heatmap with the same size as the input image. And the value of each pixel on the heatmap reflects its contribution to the final prediction of the chosen pixel.

Specifically, we choose one pixel p in the prediction image, which is predicted correctly by the network with the global-local strategy, while predicted incorrectly by the network without global-local strategy. And then, we visualize the contribution of each pixel of the input image to the prediction of p.

From our result in the Supplementary Fig. 9, the chosen points are predicted correctly by the network with the global-local strategy (red in (3)), while incorrectly by the network without global-local strategy (green in (4)). From our result, with the global-local strategy, the number of pixels that have a high contribution to the prediction is much larger than that of the network without the strategy. It suggests that the global-local strategy enables the network to utilize features of larger regions for the decision and helps improve the prediction performance.

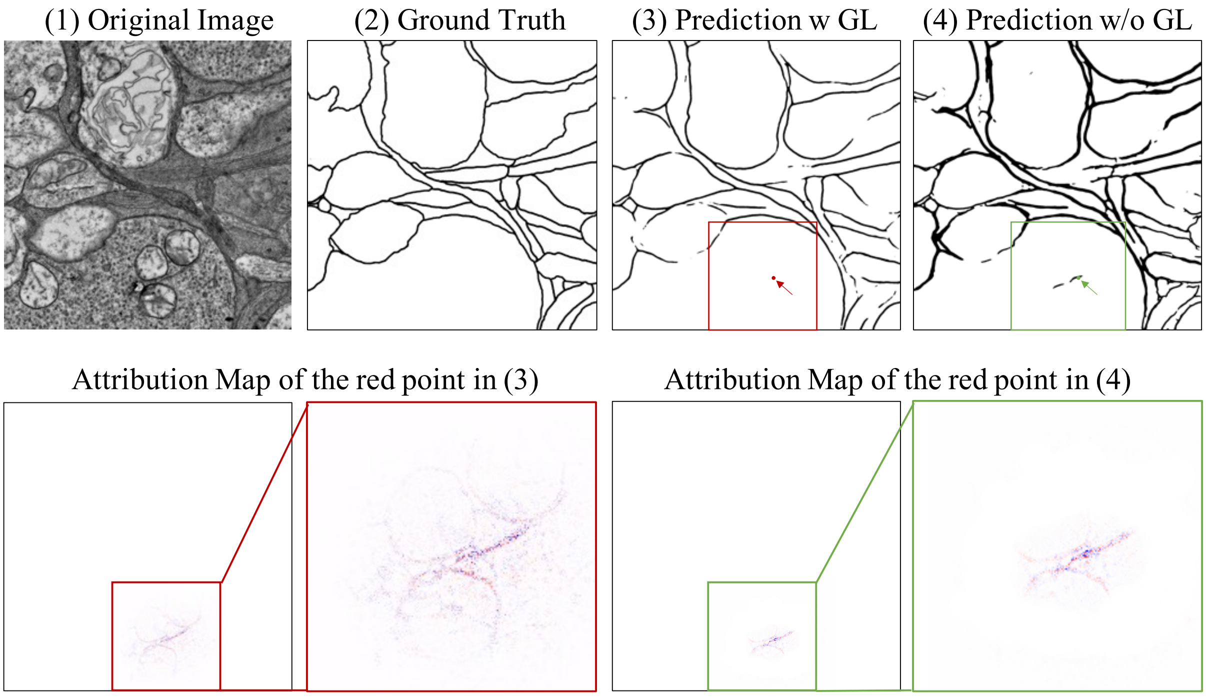

Figure 9: The images in the first row are the original image, ground truth, and two predictions with/without global-local strategy, respectively. The pixels pointed by red/green arrows are the prediction points chosen in the attribution method. The images in the second row are the attribution maps of the selected points in the two predictions. The size of the area corresponding to the same color is the same. In the attribution maps, blue means the network is likely to predict the pixels as cell membrane, while opposite for red.

7 The derivation process of the PHD loss function.

First, for the thinning process, we conducted a fully differentiable implementation for the Zhang-Suen method with PyTorch. The original Zhang-Suen skeleton method is non-differentiable due to the thresholding operations. To make it differentiable, we define a differentiable approximation to the thresholding operation used in the Zhang-Suen algorithm. We apply a soft thresholding function, ReLU, to the difference between the local intensity values and the threshold value, and use the resulting value to update the continuous function. The gradient of the ReLU function is well-defined and can be used to propagate gradients through the skeletonization algorithm. After applying the modified algorithm to the continuous function, we can obtain a continuous skeleton that is differentiable with respect to the input image.

Then, for the PHD loss, we modified the loss function. Specifically,

1) when $d(x, y) = \tau$, the gradient is not differentiable, so we set the gradient= 0 in the implementation in order to simplify mathematical calculation.

2) when $d(x, y) < \tau$, $d_{PHD}(x, y) = 0$, so the gradient is 0.

3) when $d(x, y) > \tau$, take x as an example, the formula of its gradient is

$$\begin{aligned} \frac{\partial d_{PHD}(x,y)}{\partial x} &= \frac{1}{|X|} \sum_{x \in X} \frac{\partial \min_{y \in Y} d(x,y)}{\partial x} + \frac{1}{|Y|} \sum_{y \in Y} \frac{\partial \min_{x \in X} d(x,y)}{\partial x} \\ &= \frac{1}{|Y|} \sum_{y \in Y} \frac{\partial d(x,y)}{\partial x} \end{aligned} \quad (2)$$

References

I. Arganda-Carreras, S. C. Turaga, D. R. Berger, D. Cireşan, A. Giusti, L. M. Gambardella, J. Schmidhuber, D. Laptev, S. Dwivedi, J. M. Buhmann, T. Liu, M. Seyedhosseini, T. Tasdizen, L. Kamentsky, R. Burget, V. Uher, X. Tan, C. Sun, T. D. Pham, E. Bas, M. G. Uzunbas, A. Cardona, J. Schindelin, and H. S. Seung, "Crowdsourcing the creation of image segmentation algorithms for connectomics," *Frontiers in Neuroanatomy*, vol. 9, p. 142, 2015.

R. Shi, W. Wang, Z. Li, L. He, K. Sheng, L. Ma, K. Du, T. Jiang, and T. Huang, "U-risc: An annotated ultra-high-resolution electron microscopy dataset challenging the existing deep learning algorithms," *Frontiers in Computational Neuroscience*, vol. 16, 2021.

V. Mnih, *Machine learning for aerial image labeling.* University of Toronto (Canada), 2013.

Q. Zou, Y. Cao, Q. Li, Q. Mao, and S. Wang, "Cracktree: Automatic crack detection from pavement images," *Pattern Recognition Letters*, vol. 33, no. 3, pp. 227–238, 2012.

O. Ronneberger, P. Fischer, and T. Brox, "U-net: Convolutional networks for biomedical image segmentation," in *MICCAI*, pp. 234–241, Springer, 2015.

Z. Yu, C. Feng, M.-Y. Liu, and S. Ramalingam, "Casenet: Deep category-aware semantic edge detection," in *CVPR*, pp. 5964–5973, 2017.

A. Chaurasia and E. Culurciello, "Linknet: Exploiting encoder representations for efficient semantic segmentation," in *VCIP*, pp. 1–4, IEEE, 2017.

W. Chen, Z. Jiang, Z. Wang, K. Cui, and X. Qian, "Collaborative global-local networks for memory-efficient segmentation of ultra-high resolution images," in *CVPR*, pp. 8924–8933, 2019.

J. Hu, L. Shen, and G. Sun, "Squeeze-and-excitation networks," in *CVPR*, pp. 7132–7141, 2018.

Z. Zhou, M. M. Rahman Siddiquee, N. Tajbakhsh, and J. Liang, "UNet++: A Nested U-Net Architecture for Medical Image Segmentation," in *Deep Learning in Medical Image Analysis and Multimodal Learning for Clinical Decision Support*, pp. 3–11, Springer International Publishing, 2018.

A. Mosinska, P. Marquez-Neila, M. Koziński, and P. Fua, "Beyond the pixel-wise loss for topology-aware delineation," in *Proceedings of the IEEE conference on computer vision and pattern recognition*, pp. 3136–3145, 2018.

X. Hu, Y. Wang, L. Fuxin, D. Samaras, and C. Chen, "Topology-aware segmentation using discrete morse theory," in *International Conference on Learning Representations*, 2021.

A. Paszke, S. Gross, F. Massa, A. Lerer, J. Bradbury, G. Chanan, T. Killeen, Z. Lin, N. Gimelshein, L. Antiga, *et al.*, "Pytorch: An imperative style, high-performance deep learning library," *Advances in neural information processing systems*, vol. 32, pp. 8026–8037, 2019.

A. Lou, S. Guan, and M. Loew, "Cfpnet-m: A light-weight encoder-decoder based network for multimodal biomedical image real-time segmentation," *arXiv preprint arXiv:2105.04075*, 2021.

C. Szegedy, V. Vanhoucke, S. Ioffe, J. Shlens, and Z. Wojna, "Rethinking the inception architecture for computer vision," in *Proceedings of the IEEE conference on computer vision and pattern recognition*, pp. 2818–2826, 2016.

N. Ibtehaz and M. S. Rahman, "Multiresunet: Rethinking the u-net architecture for multimodal biomedical image segmentation," *Neural Networks*, vol. 121, pp. 74–87, 2020.
M. Tan and Q. Le, "Efficientnet: Rethinking model scaling for convolutional neural networks," in *International Conference on Machine Learning*, pp. 6105–6114, PMLR, 2019.
A. Lou, S. Guan, and M. H. Loew, "Dc-unet: rethinking the u-net architecture with dual channel efficient cnn for medical image segmentation," in *Medical Imaging 2021: Image Processing*, vol. 11596, p. 115962T, International Society for Optics and Photonics, 2021.
M. Sandler, A. Howard, M. Zhu, A. Zhmoginov, and L.-C. Chen, "Mobilenetv2: Inverted residuals and linear bottlenecks," in *Proceedings of the IEEE conference on computer vision and pattern recognition*, pp. 4510–4520, 2018.
H. Zhao, X. Qi, X. Shen, J. Shi, and J. Jia, "Icnet for real-time semantic segmentation on high-resolution images," in *Proceedings of the European conference on computer vision (ECCV)*, pp. 405–420, 2018.
S. Mehta, M. Rastegari, L. Shapiro, and H. Hajishirzi, "Espnetv2: A light-weight, power efficient, and general purpose convolutional neural network," in *Proceedings of the IEEE/CVF Conference on Computer Vision and Pattern Recognition*, pp. 9190–9200, 2019.
A. Paszke, A. Chaurasia, S. Kim, and E. Culurciello, "Enet: A deep neural network architecture for real-time semantic segmentation," *arXiv preprint arXiv:1606.02147*, 2016.
Z. Gu, J. Cheng, H. Fu, K. Zhou, H. Hao, Y. Zhao, T. Zhang, S. Gao, and J. Liu, "Ce-net: Context encoder network for 2d medical image segmentation," *IEEE transactions on medical imaging*, vol. 38, no. 10, pp. 2281–2292, 2019.
W. Shen, B. Wang, Y. Jiang, Y. Wang, and A. Yuille, "Multi-stage multi-recursive-input fully convolutional networks for neuronal boundary detection," in *CVPR*, pp. 2391–2400, 2017.
T. M. Quan, D. G. Hildebrand, and W.-K. Jeong, "Fusionnet: A deep fully residual convolutional neural network for image segmentation in connectomics," *arXiv preprint arXiv:1612.05360*, 2016.
M. Lin, Q. Chen, and S. Yan, "Network in network," *arXiv preprint arXiv:1312.4400*, 2013.
H. Chen, X. J. Qi, J. Z. Cheng, and P. A. Heng, "Deep contextual networks for neuronal structure segmentation," in *Thirtieth AAAI conference on artificial intelligence*, 2016.
L. Mais, P. Hirsch, and D. Kainmueller, "Patchperpix for instance segmentation," in *European Conference on Computer Vision*, pp. 288–304, Springer, 2020.
S. Wolf, C. Pape, A. Bailoni, N. Rahaman, A. Kreshuk, U. Kothe, and F. Hamprecht, "The mutex watershed: efficient, parameter-free image partitioning," in *Proceedings of the European Conference on Computer Vision (ECCV)*, pp. 546–562, 2018.
S. Shit, J. C. Paetzold, A. Sekuboyina, I. Ezhov, A. Unger, A. Zhylka, J. P. Pluim, U. Bauer, and B. H. Menze, "cldice-a novel topology-preserving loss function for tubular structure segmentation," in *CVPR*, pp. 16560–16569, 2021.
M. Sundararajan, A. Taly, and Q. Yan, "Axiomatic attribution for deep networks," in *International conference on machine learning*, pp. 3319–3328, PMLR, 2017.
